# Predicting Iran’s achievement to Sustainable Development Goal 3.2: A systematic analysis of neonatal mortality with scenario-based projections to 2030

**DOI:** 10.1371/journal.pone.0283784

**Published:** 2023-04-06

**Authors:** Narges Ebrahimi, Sarvenaz Shahin, Sogol Koolaji, Ali Ghanbari, Parinaz Mehdipour, Masoud Masinaei, Sahar Saeedi Moghaddam, Negar Rezaei, Azin Ghamari, Mohammad-Reza Malekpour, Nazila Rezaei, Hamidreza Jamshidi, Bagher Larijani, Ardeshir Khosravi, Farshad Farzadfar

**Affiliations:** 1 Non-Communicable Diseases Research Center, Endocrinology and Metabolism Population Sciences Institute, Tehran University of Medical Sciences, Tehran, Iran; 2 Melbourne School of Population and Global Health, University of Melbourne, Parkville, Australia; 3 Department of Epidemiology and Biostatistics, Tehran University of Medical Sciences, Tehran, Iran; 4 Endocrinology and Metabolism Research Center, Endocrinology and Metabolism Clinical Sciences Institute, Tehran University of Medical Sciences, Tehran, Iran; 5 Department of Pharmacology, Research Institute for Endocrine Sciences, School of Medicine, Shahid Beheshti University of Medical Sciences, Tehran, Iran; 6 Deputy for Public Health, Ministry of Health and Medical Education, Tehran, Iran; West China Second University Hospital of Sichuan University, CHINA

## Abstract

**Background:**

Sustainable Development Goal 3.2 (SDG 3.2) is to reduce Under-5 and neonatal mortality rates (U5MR and NMR), two major health systems’ performance indicators, globally by 2030. We aimed to report Iran’s U5MR and NMR status during 2010–2017 and its achievement of SDG 3.2 by 2030, using scenario-based projection.

**Study design:**

To estimate the national and subnational levels of U5MR and NMR, we applied an Ensemble Bayesian Model Averaging (EBMA) with Gaussian Process Regression (GPR) and Spatio_temporal models. We used all available data sources including: 12-year data from the Death Registration System (DRS), two censuses, and a demographic and health surveys (DHS). This study employed two approaches, Maternal Age Cohort (MAC) and Maternal Age Period (MAP), to analyze summary birth history data obtained from censuses and DHS. In addition, we calculated the child mortality rate directly from DHS using the complete birth history method. National and subnational NMR was projected up to 2030 with a scenario-based method using average Annual Rate of Reduction (ARR) introduced by UN-IGME.

**Results:**

In 2017, national U5MR and NMR were 15·2 (12·4–18·0) and 11·8 (10·4–13·2), with an average ARR of 5·1% (2·1–8·9) and 3·1% (0·9–5·8) during 2010–2017, respectively. According to our projection scenarios, 17 provinces have not fulfilled SDG 3.2 for NMR yet, and the current trend (the current trend of NMR improvement in Iran) will not result in reaching SDG for some provinces by 2030; However, if each province has the same neonatal mortality annual reduction rate as the best-performing province in the same region, besides achieving SDG, the national NMR will be reduced to 5·2, and almost 92,000 newborn lives will be saved.

**Conclusions:**

Iran has achieved SDG3.2 regarding U5MR and NMR; however, there are provincial inequalities. For all provinces to reach SDG3.2, health policies should focus on reducing provincial inequalities by precise planning for neonatal health care.

## Introduction

Under-five Mortality Rate (U5MR) and Neonatal Mortality Rate (NMR) are essential indices for evaluating the health status and human development of countries, and have always been significant to health policy-makers [1, 2]. According to the WHO’s report in 2019, 47% of under-5 deaths occur in the neonatal period [3]. Even though U5MR has significantly decreased globally, previous studies have shown that the decline in NMR is considerably lower [4, 5].

In 2015, the United Nations adopted Sustainable Development Goals (SDGs) to improve education and health and reduce inequalities [5, 6]. According to SDG3.2, countries must lower their NMRs to 12 or lower deaths per 1,000 live births and U5MRs to 25 or lower [7]. Evaluating countries’ progress in achieving SDG3.2 requires assessing the trends of these indices through time. Iran’s Death Registration System (DRS) is incomplete, like many developing countries [8, 9]. Considering the importance of such indices in health decisions and evidence-based prioritization of health interventions and due to the low data quality for under-five and neonatal deaths in Iran, we need advanced and precise statistical models to handle data challenges and make accurate estimations of U5MR and NMR.

In this study, we conducted a comprehensive analysis to estimate U5MR and NMR. To the best of our knowledge, this is the first study in Iran that estimates NMR at national and subnational levels using all available data sources and projects it based on different scenarios.

## Methods

### Ethical approval

This study is approved by the ethics committee of “Endocrinology and Metabolism Research Institute, Tehran University of Medical Sciences, Tehran, Iran” with the following ID number: (IR.TUMS.EMRI.REC.1397.022). The data sources we used were obtained from national registries (aggregated data), and we did not interview any patients directly. Moreover, all data were anonymized before use. Thus, the confidentiality of the subjects of this study was maintained.

### Data sources

First, we identified all relevant data sources available to estimate Under-5 and neonatal mortality. Nationally representative data sources for U5MR can be obtained from different sources [10]. According to our searches, Iran’s 1996, 2000, 2011, and 2016 censuses included questions on summary birth history. The Demographic and Health Surveys (DHS) for the years 2000 and 2010 contained questions on summary and complete birth history. DRS data were also available from 1995 to 2017. To ensure the validity of our final estimates, we determined two quality criteria for our data sources before analyzing the data. First, we assessed the percentage of missing values for the key variables (a 5–10% cutoff was considered for missing values) [11, 12].

The percentage of missing values for children ever born and those who survived was calculated for summary birth history. Based on our analysis, the percentage of missing values for DHS 2010 and census 2011 were 28% and 42%, respectively. Besides, the percentage of missing values for the survival status of children, date of birth, and age at death, which are essential for the complete birth history method, were about 20%. In comparison, this amount for the same variables was less than 1% in other data sources.

Second, both DHS 2010 and census 2011 showed a sex ratio (boys-to-girls ratio) greater than 1.06; in a population, we considered a sex ratio greater than 1.06 or less than 1.00 implausible [13].

According to the sex ratio estimates at the provincial level, the majority of provinces had either a sex ratio greater than 1.06 or a sex ratio less than 1.00 in these two datasets, which differs from Iranian Statistical Center reports that state this ratio is 1.02 at the national level (varies between 1.01 and 1.06 at the provincial level) [14]. Therefore, we did not include DHS 2010 and Census 2011 in the final datasets based on these two criteria. Comparing DRS estimates with other datasets, we found that for all years between 1995 and 2005, there is a significant amount of incompleteness for DRS at the national level (more than 50%). At the sub-national level, the underestimation of death records increases and adds no unbiased data to the final estimates. As a result, we did not include DRS data from years before 2005 in the final datasets. Census 2016 also had a significant underestimation compared to other data sources, so we excluded it as well.

Consequently, we considered five measurements as primary inputs for analyzing child mortality: one summary birth history from DHS 2000, one complete birth history from DHS 2000, two summary birth histories based on censuses (1996 and 2006), and the death rates of the DRS from 2006 to 2017 (S1 File-p 4).

DHS 2000 was conducted with the participation of 113,957 households. For censuses 1996 and 2006, we used 2 percent of all population of Iran, which was 247,760 and 347,060 households, respectively. All data sources used in this study covered the population of all of Iran’s provinces [15].

Information on neonatal deaths was obtained from the DRS 2010–2017. We also used province-year-specific U5MR estimates as the explanatory variable to estimate NMR.

### Estimating under-5 mortality

This study followed two approaches, Maternal Age Cohort (MAC) and Maternal Age Period (MAP), to analyze summary birth history data obtained from censuses and DHS [16]. We analyzed complete birth history data from DHS and calculated U5MR for each year before the survey by dividing the total weighted number of under-5 deaths by the total weighted person-time of exposure (S1 File- p 5) [17].

Eventually, we had five measurements of under-5 mortalities in our analysis: summary birth histories from censuses (1996 and 2006) and DHS (2000), complete birth history from DHS (2000), and deaths from DRS during 2006–2017. We generated a single-time trend for U5MR by applying the ensemble Bayesian Model Averaging (BMA) approach to the input data mentioned. The BMA method combines posterior distributions of each ensemble model to provide final estimates [18].

The share of each model in the final estimates was determined according to its out-of-sample performance. We randomly held back %20 of DRS and %20 of data from other sources as test data. We assessed the performance of models based on Root Mean Square Error (RMSE) and %95 coverage. We calculated model weights using a monotonically declining function that uses the rank of each model [19]. This study applied an ensemble of Gaussian Process Regression (GPR) and Spatio-Temporal (ST) models to estimate U5MR.

Under the Gaussian process model, we used a Generalized Linear Mixed Model (GLMM) with relevant covariates (including wealth index, years of schooling, and urbanization) to estimate U5MR for the mean function. The information for wealth index and years of schooling was obtained from Household Income and Expenditure survey. These data are gathered annually by the Statistical Center of Iran using a standard questionnaire [20]. Moreover, urbanization rates were calculated using censuses (S1 File-p 11).

Then, we calculated adjacency matrices and borrowed strengths over provinces and years using GLMM residuals. The Matérn function, as the covariance function, uses parameters to control the time trend’s correlation and smoothness. We also considered the incompleteness of DRS and both sampling and non-sampling errors in the GPR model (S1 File- p 10). We generated samples from the posterior distributions of model parameters and U5MRs using the Markov Chain Monte Carlo (MCMC) method within the R-Stan package, version 3.2.0 [21].

As the next model, we applied a spatiotemporal model in a Bayesian framework, containing three components: spatial, temporal, and spatiotemporal interaction terms. We used the Besag-York-Mollie (BYM) correlation structure, a conditional autoregressive (CAR) model, to consider the spatial dependency in U5MRs. Besides, we imposed a random walk of order 2 to address the temporal dependency. All analyses were conducted using R-INLA, version 3.2.0 (S1 File- p 16) [22, 23].

GPR provided U5MR estimates by considering the difference between data sources, the incompleteness of DRS, and both sampling and non-sampling errors. However, the spatiotemporal model considers spatial correlation strongly in estimating U5MRs. Finally, we used the BMA approach to combine the GPR and spatiotemporal models to take advantage of both models (S1 File- p 18).

### Estimating neonatal mortality

The logarithmic NMR was modeled as a function of U5MR and province-year-specific effect [24]. We found that the global association between log (NMR) and log (U5MR) was quadratic (S1 File- p 21). The province-year-specific effects allow provinces to have higher or lower NMR than the expected NMR based on U5MR. We also used a truncated distribution to ensure that NMR does not exceed U5MR. To model the province-year-specific effects, we used both GPR and spatiotemporal models. The GPR considered the relation of NMR and U5MR in its mean function. Similar to the estimation of U5MR, we employed the BMA technique to combine both models (S1 File- p 20).

### Scenario-based projections

We used five scenarios for projecting NMRs from 2018 to 2030 at the national and subnational levels [6]. Neonatal mortality was projected according to a constant or a declining NMR employing an annual rate of reduction (ARR), which was defined as $ARR=\frac{\text{log}\left(NMR_{t_{2}}/NMR_{t_{1}}\right)}{\left(t_{1}-t_{2}\right)}$, that t1 and t2 are years (t1< t2). Each scenario is fully explained in the results section.

## Results

### Under-5 mortality rates

**Fig 1:**
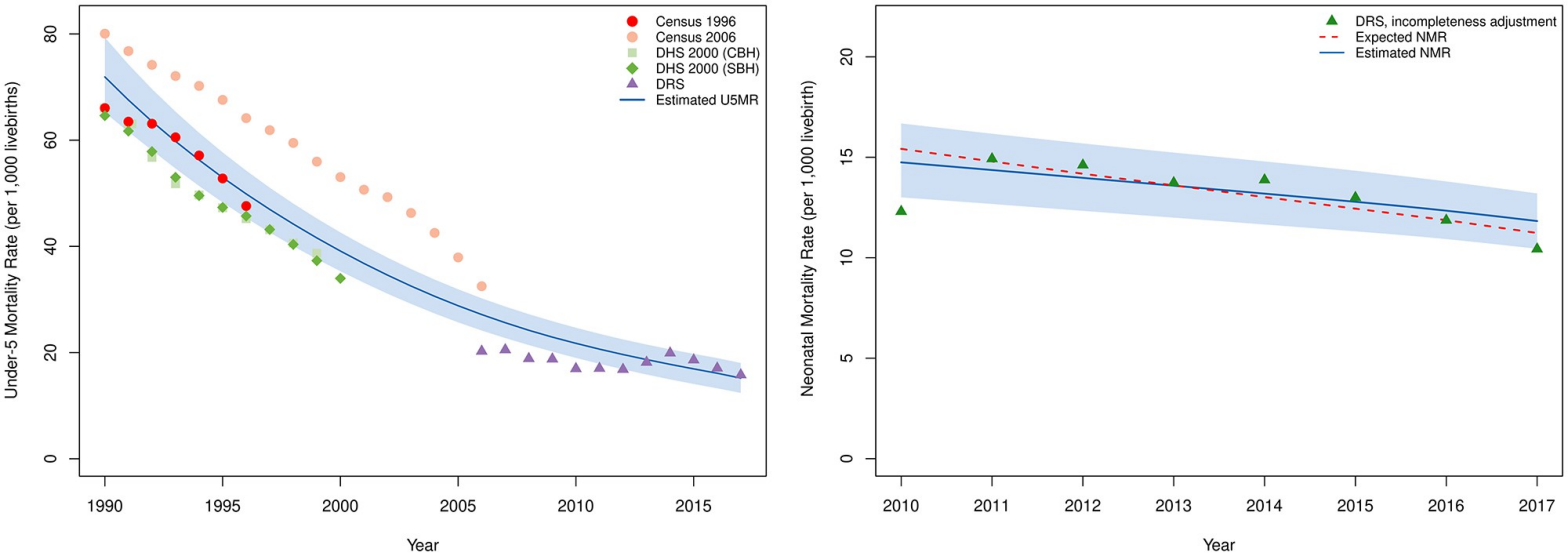
National trends of U5MR and NMR before 2017.

At the national level, U5MR in Iran decreased from 21·7 (95%CI 19·0–24·7) deaths per 1,000 livebirths in 2010, to 15·2 (95%CI 12·4–18·0) in 2017. This 30% reduction lowered the number of annual under-5 deaths from 29,971 (95%CI 26,188–34,010) to 24,409 (95%CI 19,927–28,978) during this period. The Average ARR of U5MRs (U5M-AARR) was 5·1% (95%CI 2·1–8·9) between 2010 and 2017 (Table 1, Fig 1).

**Table 1:** National, regional, and provincial U5MRs and NMRs in 2010 and 2017, with AARRs during 2010–2017.

	Under-5mortality*			Neonatal mortality*		
	U5MR (Death per 1,000 livebirths)		Average ARR (%)	NMR (Death per 1,000 livebirths)		Average ARR(%)
	2010	2017	2010–2017	2010	2017	2010–2017
**National**	21.7(19.0–24.7)	15.2(12.4–18.0)	5.1(2.1–8.9)	14.7(13.0–16.7)	11.8(10.4–13.2)	3.1(0.9–5.8)
**West Region**	22.6(20.0–25.2)	15.6(12.6–17.8)	5.3(1.5–9.5)	15.5(13.7–17.6)	12.2(10.8–13.6)	3.4(0.9–5.9)
**North and Northeast Region**	21.7(19.3–24.6)	15.0(12.9–18.1)	5.3(1.6–10.3)	13.9(12.5–16.0)	11.2(10.0–12.7)	3.1(0.8–5.5)
**Southeast Region**	26.5(23.4–29.8)	18.1(14.8–21.4)	5.4(1.6–9.9)	18.0(15.6–20.0)	14.9(12.7–16.1)	2.7(0.4–4.8)
**Central Region**	18.3(15.2–21.4)	13.3(10.5–16.7)	4.6(0.3–9.0)	12.8(11.1–14.3)	10.1(9.1–11.5)	3.4(1.1–5.6)
**Alborz**	16.4(15.3–19.2)	11.8(10.5–14.4)	4.7(1.8–7.3)	10.8(9.8–12.6)	8.5(8.0–10.0)	3.5(1.0–5.7)
**Ardabil**	19.3(18.0–22.4)	12.0(10.0–13.5)	6.8(4.9–10.3)	13.5(11.9–15.5)	10.3(8.3–11.2)	3.8(2.2–7.7)
**Azerbaijan, East**	20.9(17.6–24.7)	13.1(10.2–17.9)	6.6(1.5–11.0)	12.8(12.2–15.6)	9.3(8.6–10.9)	4.5(2.4–7.4)
**Azerbaijan, West**	26.9(22.8–28.5)	15.8(13.1–17.3)	7.6(5.2–10.5)	18.3(16.3–21.4)	13.1(11.4–14.1)	4.8(3.0–7.6)
**Bushehr**	23.0(19.3–24.7)	17.7(13.8–19.3)	3.7(0.8–7.5)	15.4(13.7–17.3)	13.7(12.1–15.2)	1.7(-0.6–4.0)
**Chahar Mahaal and Bakhtiari**	17.5(15.0–19.1)	11.7(9.3–13.7)	5.7(2.5–9.0)	11.1(10.0–13.0)	8.3(7.6–9.5)	4.2(2.0–7.0)
**Fars**	24.5(23.1–28.0)	19.9(15.7–22.3)	3(1.3–7.1)	15.9(14.2–18.1)	14.4(13.2–16.7)	1.4(-1.6–3.3)
**Gilan**	12.7(9.8–15.2)	7.2(5.9–9.4)	8(2.5–11.8)	9.2(7.8–10.0)	5.2(4.4–5.8)	8.2(5.8–10.8)
**Golestan**	18.9(16.3–19.7)	12.4(9.7–14.1)	6.1(3.0–9.5)	12.7(11.7–14.7)	10.4(8.5–11)	2.8(1.8–7.0)
**Hamadan**	23.2(20.5–24.9)	16.0(12.9–17.2)	5.3(3.3–8.6)	18.4(15.1–19.4)	14.1(11.9–14.8)	3.9(1.5–5.6)
**Hormozgan**	27.3(22.8–31.5)	19.9(15.3–24.2)	4.6(0.4–8.7)	19.1(16.2–20.5)	16.2(13.9–17.6)	2.3(-0.1–4.4)
**Ilam**	23.0(19.3–25.7)	17.1(13.4–21.3)	4.2(0.0–7.9)	17.1(14.5–18.7)	14.2(12.5–15.6)	2.6(0.1–4.6)
**Isfahan**	20.3(17.2–21.7)	16.3(12.2–18.6)	3.2(0.2–7.4)	14.9(12.4–16.0)	12.8(11.3–14.2)	2.2(-0.9–3.4)
**Kerman**	22.5(20.6–25.5)	17.1(14.5–19.2)	4(2.1–7.1)	15.1(13.7–17.4)	15.1(12.1–15.7)	0.1(-0.8–4.5)
**Kermanshah**	24.5(21.6–26.0)	18.2(14.9–19.5)	4.2(2.2–7.0)	19.3(16.2–20.3)	16.0(13.9–16.8)	2.7(0.2–4.5)
**Khorasan, North**	28.4(24.7–33.2)	18.2(14.3–26.9)	6.3(0.4–10.3)	17.2(15.5–20.0)	13.0(11.9–15.4)	4.1(1.4–6.1)
**Khorasan, Razavi**	26.5(24.2–30.7)	18.5(16.6–22.0)	5.1(2.4–7.7)	16.4(15.0–19.1)	13.6(12.3–15.6)	2.7(0.3–5.4)
**Khorasan, South**	31.3(25.5–37.2)	18.8(14.4–23.3)	7.3(3.3–11.3)	20.2(17.6–22.7)	14.7(13.2–16.4)	4.6(2.2–6.8)
**Khuzestan**	19.8(18.7–23.0)	14.3(12.3–16.2)	4.6(2.9–7.7)	13(11.9–15.3)	11.1(9.8–12.3)	2.3(0.6–5.2)
**Kohgiluyeh and Boyer-Ahmad**	22.7(19.0–25.1)	15.8(12.5–17.8)	5.1(2.2–9.0)	13.8(12.7–16.2)	11.1(10.3–13.2)	3.1(0.4–5.2)
**Kurdistan**	29.2(24.5–30.7)	18.9(14.7–20.4)	6.3(3.6–9.7)	21.0(17.8–23.2)	15.9(14.0–17.2)	4(1.8–6.0)
**Lorestan**	18.5(15.5–19.7)	11.8(9.2–13.4)	6.4(3.0–9.8)	14.2(11.4–15.1)	9.0(8.0–10.2)	6.5(2.7–7.7)
**Markazi**	21.1(18.0–25.3)	14.4(11.4–19.2)	5.5(0.5–9.6)	17.4(13.7–18.4)	12.9(10.6–13.6)	4.3(1.7–6.2)
**Mazandaran**	13.8(11.9–14.6)	9.6(7.8–10.6)	5.2(2.6–8.1)	9.5(8.4–10.8)	7.1(6.2–8.0)	4.1(1.7–6.2)
**Qazvin**	19.7(18.3–22.9)	14.3(12.8–16.9)	4.6(1.8–7.4)	14.2(12.2–15.5)	11.4(10.1–12.5)	3.1(0.7–5.4)
**Qom**	15.5(14.4–17.4)	9.5(8.2–10.8)	7(5.0–9.9)	11.6(9.7–12.6)	7.4(6.3–8.0)	6.4(3.9–8.5)
**Semnan**	22.2(20.2–24.6)	15.4(13.3–17.5)	5.2(2.9–7.9)	15.6(13.1–17.3)	12.1(10.5–13.4)	3.6(1.2–5.7)
**Sistan and Baluchistan**	27.9(25.1–30.7)	17.8(14.8–21.1)	6.5(3.7–9.7)	19.1(16.3–21.0)	14.2(12.4–15.7)	4.3(1.9–6.3)
**Tehran**	16.9(13.6–20.7)	12.1(9.6–16.2)	4.7(0.2–9.4)	11.4(10.3–13.1)	9.0(8.2–10.4)	3.3(0.5–5.5)
**Yazd**	23.2(15.6–27.7)	16.4(10.5–20.6)	4.9(-1.6–11.8)	14.9(13.2–17.2)	12.1(11.0–13.9)	3(0.7–5.3)
**Zanjan**	24.0(21.4–28.6)	14.5(12.5–17.3)	7.2(4.4–10.7)	16.3(14.6–18.7)	11.4(10.2–12.9)	5.1(2.9–7.7)

*All rates are reported with 95%CI

**Fig 2:**
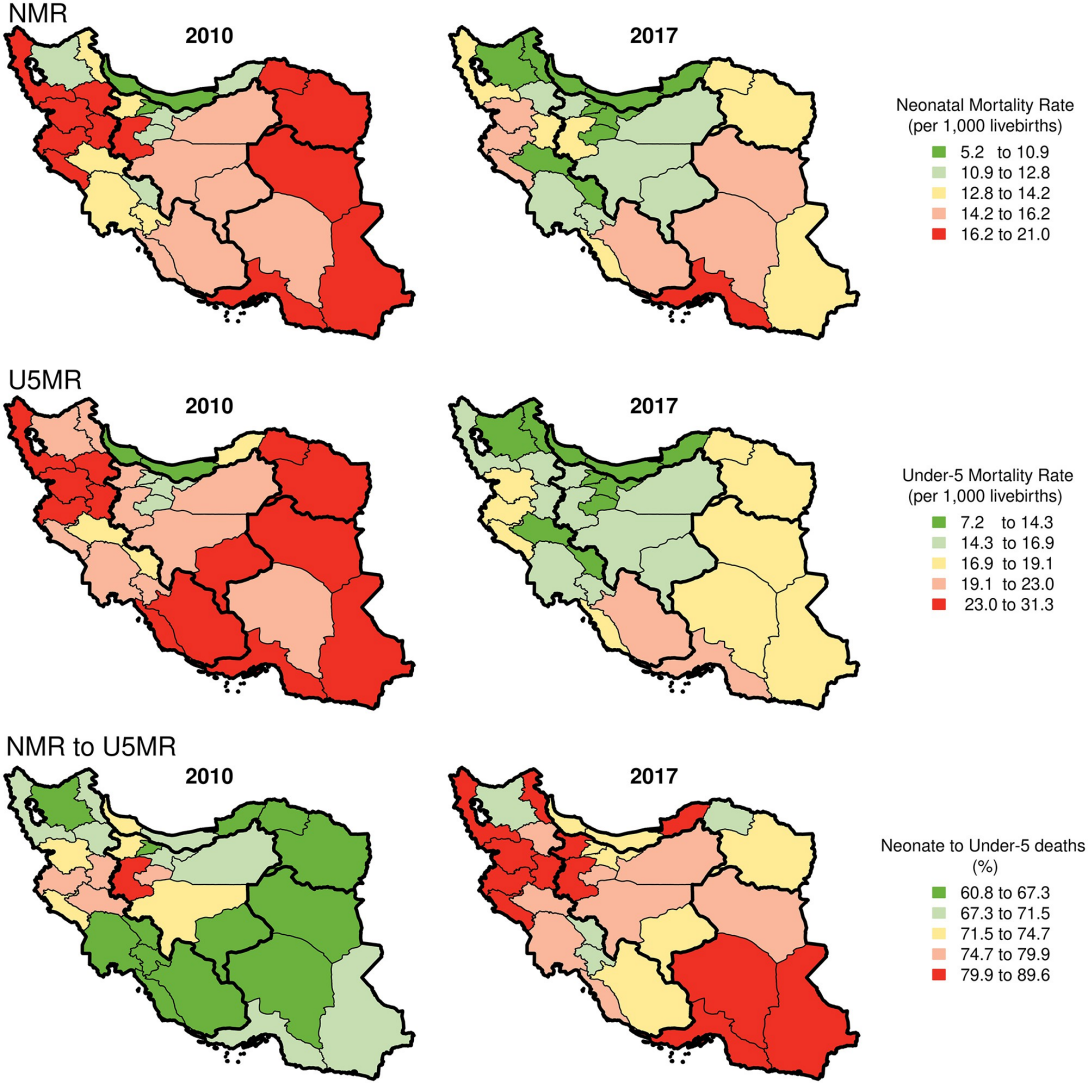
Map of the provincial U5MRs, NMRs, and neonatal share of under-5 mortality rates in 2010 and 2017 (contains information from OpenStreetMap and OpenStreetMap foundation, which is made available under the open database license).

Across the four regions of Iran [25], the mean U5MR was highest (26·5 [95%CI 23·4–29·8]) in the southeast region and lowest (18·3[95%CI 15·2–21·4]) in the central region in 2010. A similar pattern was observed in 2017 with U5MRs of 18·1 (95%CI 14·8–21·4) and 13·3 (95%CI 10·5–16·7) in southeast and central regions, respectively (Table 1). The highest provincial U5MRs are concentrated in the south, east, and some provinces of the west part of the country. The Northside provinces had the lowest U5MRs in 2010, and this pattern spread through the northwest and central parts until 2017 (Fig 2).

The difference between the highest and lowest provincial U5MRs was 18·6 (12.5–25.4) in 2010, which converged to 12·7 (8.1–15.5) in 2017; however, not statistically significant. The highest provincial U5MRs in 2017 were observed in two southern provinces (Hormozgan; 19·9 [95%CI 15·7–22·3] and Fars; 19·9 [95%CI 15·3–24·2]), where Gilan, a northern province, had the lowest UM5R (7·2 [95%CI 5·9–9·4]). All provinces showed decreasing U5MR trends during the study period.

**Fig 3:**
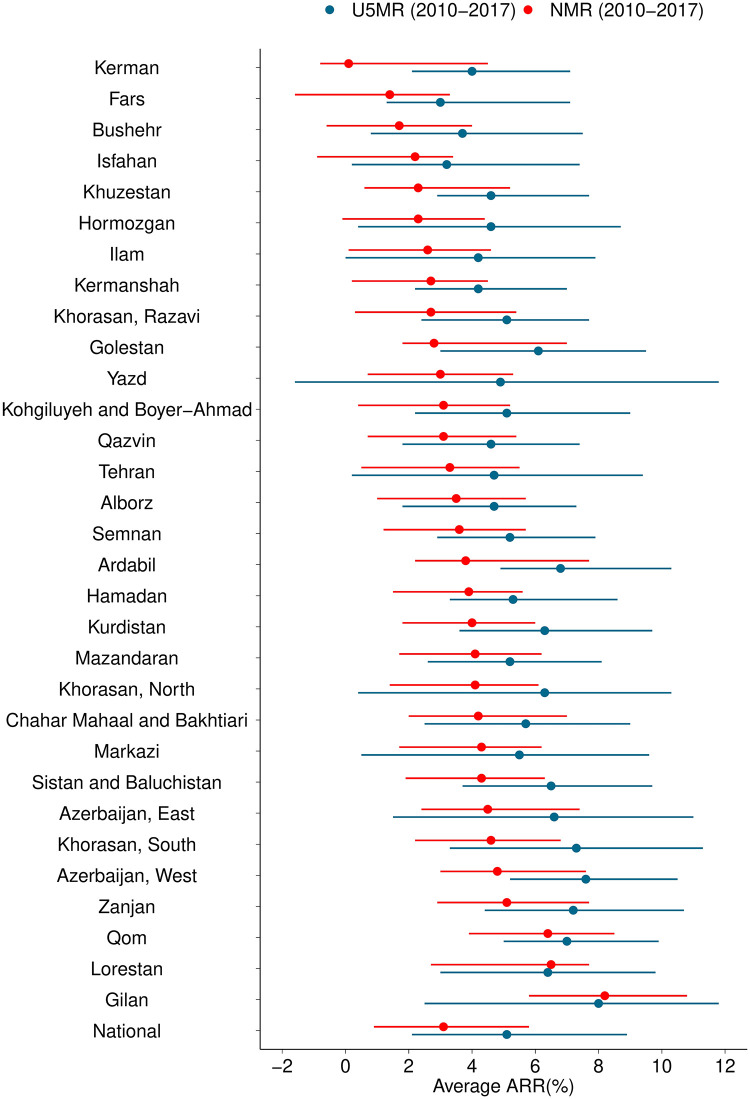
National and provincial U5M-ARRs and NM-ARR between 2010 and 2017.

The most notable reduction of U5MR was seen in South Khorasan, which changed from 31·3 (95%CI 25·5–37·2) in 2010 to 18·8 (95%CI 14·4–23·3) in 2017 (Fig 3). U5MR reduction rates in 2010–2017 ranged from 3% to 8% among different provinces (Table 1).

### Neonatal mortality rates

National NMR decreased from 14·7 (95%CI 13·0–16·7) to 11·8 (95%CI 10·4–13·2) during the study period. In 2010, 20,329 (95%CI 17,927–23,003) neonatal deaths occurred, while 18,986 (95%CI 16,764–21,199) neonates died in 2017. In the study period, the national average ARR of NMR (NM-AARR) was 3·1% (95%CI 0·9–5·8). In 2017, a wide range of NMRs was observed throughout the country, with the highest provincial NMR being over 3-fold higher than the lowest (Table 1, Fig 1).

The regional and provincial pattern of NMRs is similar to those of U5MRs. In 2010, the southeast region had the highest (18·0 [95%CI 15·6–20·0]) mean NMR, whereas the central region had the lowest (12·8 [95%CI 11·1–14·3]). This pattern continued up to 2017, in which NMRs were 14·9 (95%CI 12·7–16·1) and 10·1 (95%CI 9·1–11·5) in the southeast and central regions, respectively (Table 1). Similar to U5MRs, the provinces with the lowest NMRs were located in the northern and some parts of the central regions in 2010 (Fig 2). The same pattern can be seen in 2017, although almost all northern and central provinces improved their NMRs to the lowest categories.

The difference between the highest and lowest provincial NMRs was 11·8 (8.5–14.4) in 2010, and converged to 11·0 (8.9–12.4) in 2017, although not significantly. The highest provincial NMRs were observed in Kurdistan (21·0 [95%CI 17·8–23·2]) and South Khorasan (20·2 [95%CI 17·6–22·7]) in 2010. However, in 2017 Hormozgan became the province with the highest NMR, with 16·2 (95%CI 13·9–17·7) deaths. Gilan province had the lowest NMRs, and reduced it from 9·2 (95%CI 7·8–10·0) to 5·2 (95%CI 4·4–5·8) during this period.

**Fig 4:**
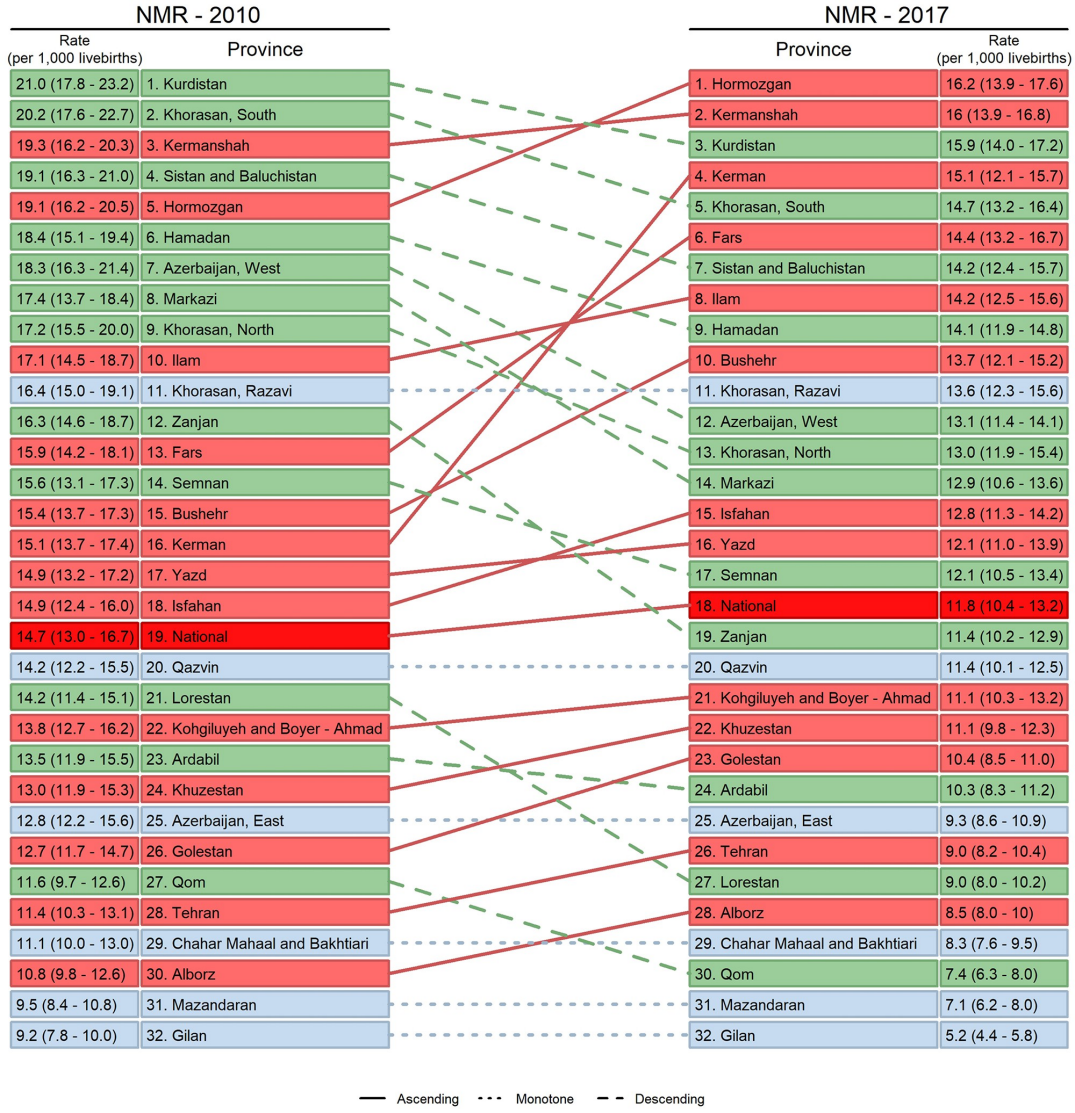
Provincial NMR ranks in 2010 versus 2017.

All provincial NMRs had decreasing trends, although with a vast range of AARRs, from Kerman with NM-AARR = 0·1% (95%CI -0·8–4·5) to Gilan with NM-AARR = 8·2% (95%CI 5·8–10·8) (Table 1, Fig 3). The decrease in 5 provinces (Kerman, Fars, Bushehr, Isfahan, and Hormozgan) was not statistically significant. Despite the remarkable drop in South Khorasan’s NMR (from 20·2 [95%CI 17·6–22·7] to 14·7 [95%CI 13·2–16·4]), it was still among the provinces with relatively high rates in 2017 (Fig 4).

### Neonatal proportion of U5MR

**Fig 5:**
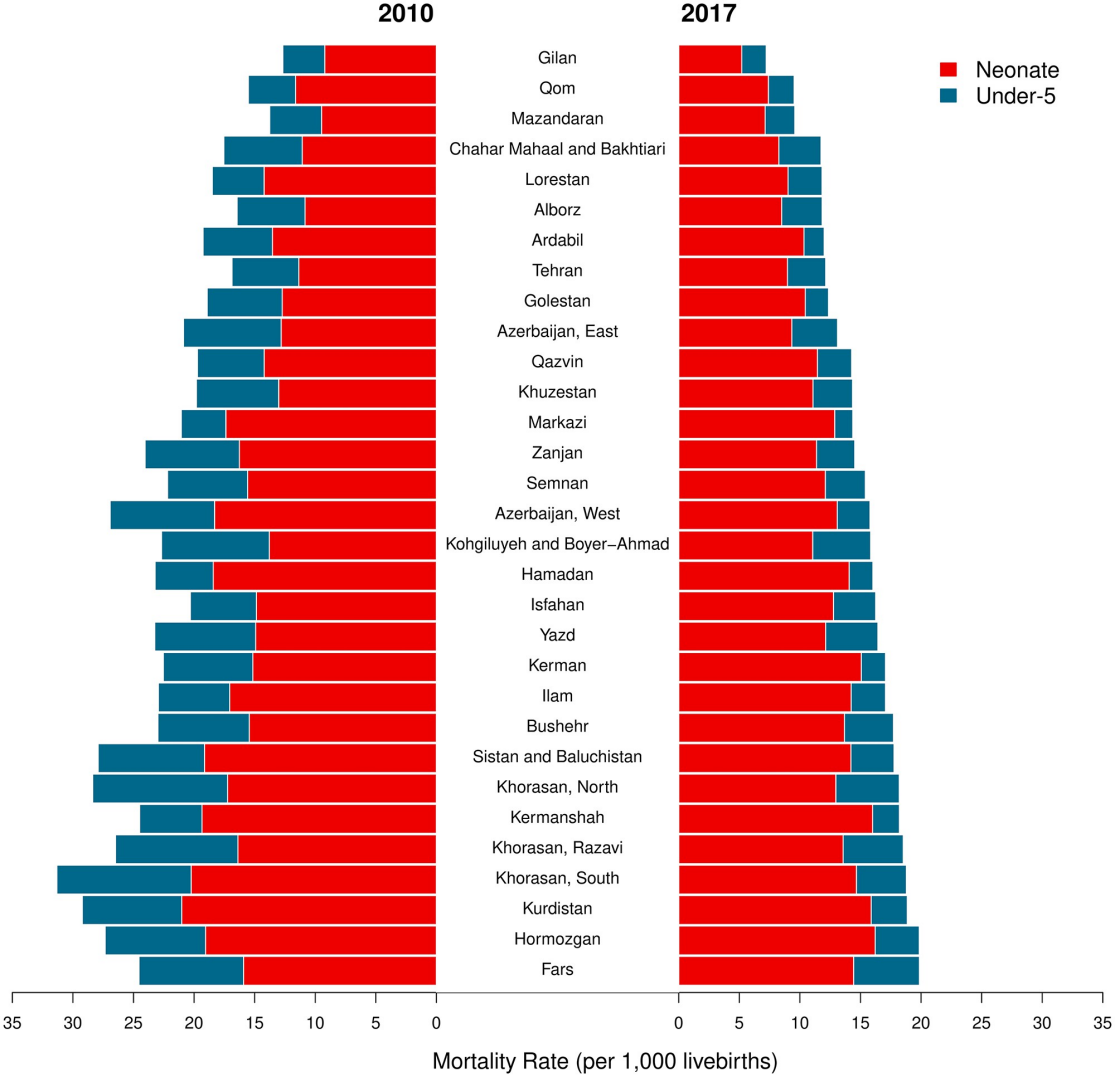
Provincial neonatal share of under-5 mortality rates in 2010 versus 2017.

The neonatal proportion of U5MR in Iran was 67·8% (95%CI 56·8%-81·3%) in 2010, and rose to 77·7% (95%CI 62·6%-97·4%) in 2017. This proportion was above 70% in all provinces in 2017, ranging from 70·1% (95%CI 60·3%-91·8%) to 89·1% (95%CI 63·4%-100·0%). All provinces showed an increasing trend in this proportion, except for Gilan and Lorestan, which remained almost stable. However, these changes were not statistically significant. A remarkable increase was observed in Kerman’s proportion, from 67·2% (95%CI 57·4%-77·7%) to 87·9% (95%CI 70·5%-100·0%). This is due to its U5MR reduction of 24%, while its NMR remained the same (Fig 5).

### Neonatal mortality projection scenarios

**Fig 6:**
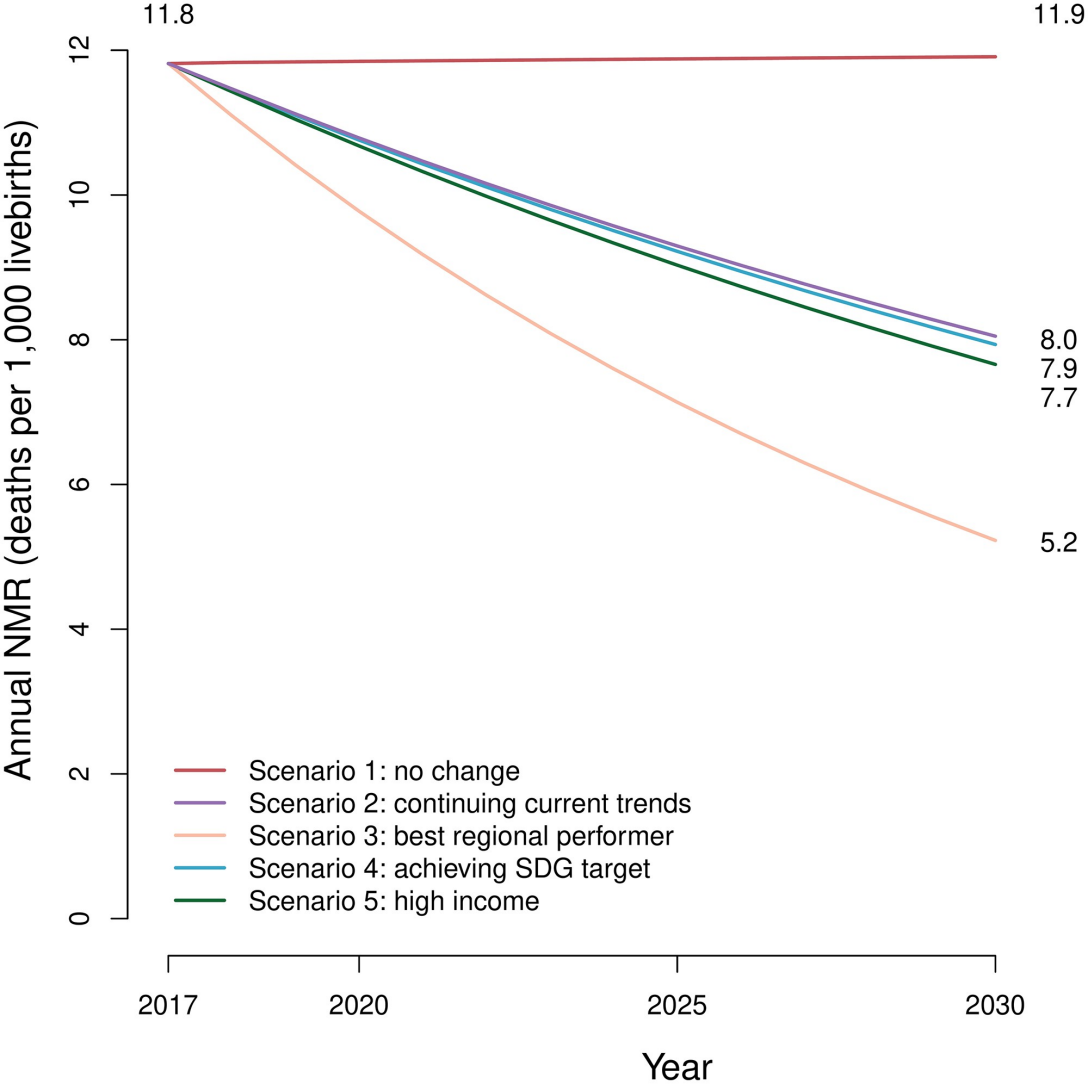
National NMR projection to 2030 based on five scenarios.

We projected neonatal mortality trends at national and provincial levels by five different scenarios from 2018 to 2030 (Fig 6, S1 File- p 38).

**Fig 7:**
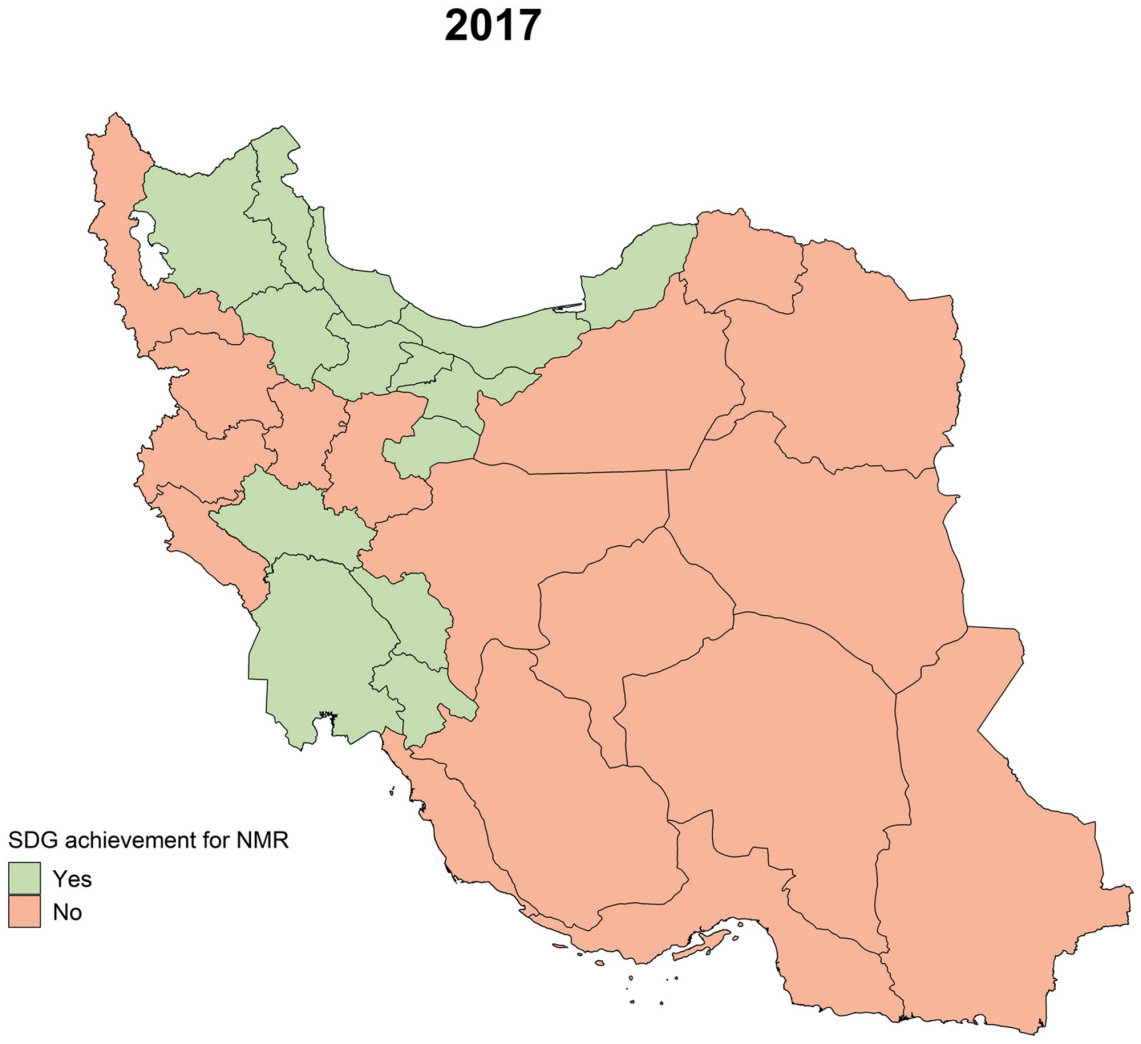
NMR SDG 3.2 achievement of 31 provinces in 2017 (contains information from OpenStreetMap and OpenStreetMap foundation, which is made available under the open database license).

In the first scenario, if national NMR in 2030 remains the same as in 2017 (NMR = 11·8), 263,117 neonatal deaths will happen during 2018–2030, with 21,850 of them in 2030. According to this scenario, in 2030, the NMRs of 17 provinces will still be higher than the SDG target (Fig 7).

The second scenario assumes that the NM-ARR of 2018–2030 remains the same as the NM-ARR during 2010–2017; therefore, the annual NMR will be lowered to 8·04 by 2030, equal to 14,765 deaths. The cumulative number of neonatal deaths during this period is estimated to be 212,586. Based on this scenario, three provinces will not reach the SDG target by 2030.

In the third scenario, the NM-ARR of each province during 2018–2030 should be equal to the NM-ARR of the best-performing province in its region from 2010 to 2017. Following this pattern, 9,588 neonatal deaths are predicted to happen in 2030, equal to 5·2 deaths per 1,000 live births. Also, the cumulative number of deaths will be 171,276. Hence, this scenario ends in a significantly lower predicted NMR than all others, and almost 92,000 neonates will potentially be saved compared to the first scenario. Besides, all of the provinces will fulfill the SDG target by 2030.

In the fourth scenario, SDG is considered the target; thus, the NMR should be lowered to 12 or fewer deaths per 1,000 live births by 2030. Based on this scenario projection, the national NMR is estimated to be 7·9 in 2030. The total and 2030 numbers of deaths will be 211,122 and 14,555, respectively.

If we consider the NM-ARR of the highest-income provinces as the goal, the fifth scenario will happen. The number of neonatal deaths and NMR are estimated to be 14,050 and 7·65 in 2030. With this scenario, 207,415 neonatal deaths will happen in 2018–2030. Similar to the third scenario, all provinces will reach the SDG target, although the national NMR will be greater.

To sum up, although the fifth one is predicted to end in lower NMR than the fourth and the second, the difference is not statistically significant.

## Discussion

We studied Iran’s U5MR and NMR during 2010–2017 based on all available measurements to assess its achievement of SDG 3.2. Iran has significantly improved these measures, and has reduced U5MR to 15·2 and NMR to 11·8 by 2017, thus has fulfilled the target in both aspects. Nevertheless, we present two significant points that we faced: First, the discrepancy between the provinces causing an NMR range of 5·2 to 16·2 throughout the country, and second, the matter that the NMR reduction rate has not been as desirable as U5MR reduction, and consequently, the neonatal proportion of U5MR has remarkably increased to 77·7%.

Global Burden of Diseases (GBD) and the United Nations Interagency Group for Child Mortality Estimations (UN-IGME), two key resources that provide estimations on such measures globally, support our results in Iran’s achievement of SDG 3.2 and the declining trends of NMR and U5MR [6, 26]. However, UN-IGME estimates of NMRs were limited to the national level, and GBD did not consider DRS as an important dataset in its estimates of NMRs at the provincial level.

Many determinants have previously been mentioned in the literature for neonatal and under-five mortalities [27, 28]. Since most (77·7%) of our U5MR is attributable to neonatal deaths, we discuss some neonatal mortality determinants here. These determinants can be classified into health-related and non-health-related factors. The most frequently mentioned non-health determinants are education and literacy (especially in women of reproductive age and mothers), mother’s age, family planning (birth interval control and lower total fertility rates), insurance coverage, and socioeconomic status, which directly or indirectly affect other determinants crucially [6, 29].

Regarding the health-related determinants, Reproductive, Maternal, Newborn, and Child Health (RMNCH continuum of care) services to reduce preterm labour (PTL), low birth weight (LBW), and congenital anomalies (the major contributors to neonatal death [30]) have been mentioned in the literature [6, 30, 31]. Examples of such services are primary health services for antenatal and newborn care, skilled birth attendants and facility delivery, life-saving interventions for mothers and children, and care for small and sick newborns. These services’ coverage, quality, and utilization are essential factors that can maximize their effectiveness [29, 32]. It is noteworthy that health and non-health determinants cannot be entirely separated since much interaction exists among these two groups. For instance, socioeconomic status can undeniably affect the access to, and the quality of health services received in many settings, or families’ higher literacy would possibly lead them to seek medical care, thus having higher utilization rates in the country.

Iran’s decreasing trends in NMR and U5MR, and its achievement of SDG 3.2 can be attributable to its advances in these determinants during the past decades. The total literacy rate has risen outstandingly since 2000 [33]. The number of illiterate young women (per thousand) almost halved from 2008 to 2016 [34]. Moreover, urbanization, which has been shown to improve access to healthcare and, thus, lower child mortality [33], has increased in Iran’s provinces in recent years [35]. In addition to these social improvements, health interventions have also been done. Iran has implemented many RMNCH interventions during the past two decades, namely well-baby care, integrated mother’s healthcare, baby-friendly hospitals, and Integrated Management of Childhood Illnesses (IMCI) [36]. The rate of skilled birth attendance has increased markedly in Iran, which according to previous studies, is an important contributor to NMR improvement [36]. Another effective program that has provided better access to health services in Iran is the Family Physician Program since 2005. In this program, family physicians and midwives provide free primary reproductive, maternal, and neonatal services for the population [37]. This program has been impressively successful, although some aspects, such as the quality of the care provided, are yet to be improved [37].

Despite all advances stated earlier, we encountered two noteworthy matters in our study; the remarkable rise in the neonatal proportion of U5MR, and the fact that there is inequality in NMR and U5MR among provinces, and not all of our provinces have fulfilled SDG3.2.

Regarding the increase in the neonatal proportion of under-5 deaths, this pattern is not limited to Iran, and similar trends have been observed in other regions. A study mapping neonatal and child mortalities in 99 low- and middle-income countries (LMICs), showed that from 2000 to 2017, this proportion rose from 37·4% to 43·7% [38]. This can probably be attributable to the fact that routine national interventions have an enormous impact on child mortality [38–40]. Improving sanitation, routine childhood vaccination, and effective treatment of infectious diseases that have been an important cause of under-5 death [27, 28], are simple and relatively low-cost though high-impact interventions [38], when compared to the interventions needed for addressing neonatal mortality contributors. According to previous estimations, Iran has succeeded in reducing U5MRs remarkably, considering the mentioned factors [10]. Thus, this can contribute to a significantly high neonatal proportion of U5MRs in Iran.

Most neonatal deaths have been shown to be related to complications associated with PTL, LBW, and also intrapartum events (such as birth asphyxia) [6, 30]. Literature shows that the PTL rate is relatively high in Iran [41], and to diminish this rate, underlying causes need to be addressed. There are many underlying factors that might lead to PTL, including not receiving proper pregnancy care, unwanted pregnancy, mother’s obesity or underweight, low socioeconomic status, etc. [30]. Addressing these root causes to improve PTL rate (as the most important contributor to NMR in the last decade [6]) and thus, reducing neonatal mortality will possibly need much effort, and will be time-consuming and costly. Moreover, in a study from Lancet Every Newborn Series, the most effective interventions to reduce NMR are stated as “Care during labour and birth”, “Immediate newborn care”, and “Care for small and sick newborns” [31]. National coverage of these packages will need resources, infrastructures, and facilities (qualified hospital and delivery facility staff, Neonatal Intensive Care Units (NICUs), etc.). Additionally, many studies have shown that only the existence of these interventions would not suffice, and the care quality plays a crucial role [6, 29, 30, 42]. Lohela et al. studied neonatal mortality in 72 LMICS, and showed that facility deliveries and care for sick newborns did not alter NMRs significantly, as long as the care quality is not desirable [32]. However, quality improvement often faces challenges and lacks well-defined strategies [43]. Overall, having affordable, high-quality RMNCH services for every woman and newborn, and improving their utilization of such services is the key to more desirable decreasing trends of NMR, which requires considerable effort and time.

Considering the observed discrepancy throughout our country, despite the reduction in overall inequality, in some of the provinces, NMR was over three times more than some others. Seventeen provinces still need to achieve the neonatal part of SDG 3.2 target, and according to our projections, 3 of them will not fulfill the target by 2030, if the same trend continues. This is while some other provinces already have much lower than SDG 3.2 NMRs. This inequality has previously been observed in other studies [10, 44]. Most of the provinces with undesirable rates were lower-income provinces and were either located at the borders or were southern provinces. These provinces share similar features, such as a low Human Development Index (HDI) [45], and a lower concentration of healthcare workers [34, 46]. Urbanization is significantly lower in these provinces [35]. Moreover, a study assessing pregnant women’s health literacy manifested unfavorable health literacy levels in pregnant women in the south of Iran [47]. In order to generally reduce the discrepancy between our provinces, future attempts can possibly focus on the above-mentioned factors.

According to our scenario-based projections, the most promising target is to improve each provincial NM-ARR to the same rate as the best-performing province in its geographical region. In order to alter the current trends more effectively, considerable effort and investment are needed to ensure every pregnant woman (and her newborn) has access to life-saving interventions, quality primary RMNCH services, and high-quality special care for small and sick newborns [6]. However, one significant matter which has recurrently been mentioned as a determinant of child and neonatal mortality is socioeconomic status and wealth index [29, 44, 48], and the factors that these indices determine, such as nutrition [30]. Poverty remarkably impacts these rates, since it affects the population’s health and non-health risk factors. Regardless of all actions taken to reduce neonatal mortality rates in Iran, the current economic status does not show promising trends. The economic burden due to the COVID-19 pandemic and the sanctions against Iran has caused inflation to surge to over 48%, and according to World Bank, poverty has reached 20% in Iran as of March 2021 [49]. These financial uncertainties might possibly slow Iran’s progression towards achieving optimal scenarios and having SDG 3.2 fulfilled for all provinces.

There are some limitations to this study. Since DRS was the only available data source for estimating the neonatal mortality rate in this period and there were no additional data sources to calculate DRS’s incompleteness on neonatal death, we had to consider DRS’s incompleteness for under-five death as a proxy of incompleteness for neonatal death. Moreover, there are limited studies in Iran investigating the factors related to neonatal health at the subnational levels during the study period. Thus, we cannot definitively compare NMR results based on neonatal health factors. Our study contains remarkable strengths: 1) we benefited from the maximum reliable data reported by responsible organizations in Iran; 2) we utilized an ensemble BMA model to outperform separate GPR and ST models in all aspects, including prediction accuracy and bias reduction. A major challenge we tried to address was that there is no standard and comprehensive neonatal and child mortality registry in Iran to cover the past 30 years; therefore, we used a variety of data sources and complex statistical methods to aggregate the data points.

Additionally, since there are many aspects to predicting future NMR trends and myriads of events can affect it, this prediction would be a complicated task. Therefore, our main goal was to provide a method for policymakers to be able to roughly predict their trends and determine which scenario they would want to base their policies on during the next few years. It is in fact necessary for anyone using this method to update the estimations on a regular basis.

Iran had favorable declining trends of U5MR and NMR, and has generally achieved SDG 3.2. Nonetheless, there is still inequality among different provinces. Moreover, since the neonatal proportion of U5MR has increased markedly in Iran, special attention needs to be given to NMR determinants. According to our projections, although current decreasing trends are towards NMR improvement, three provincial NMRs will still remain above SDG 3.2 target until 2030. However, if each province operates as the best-performing province in its region, the most suitable outcomes will be attained, and over 90,000 lives will potentially be saved by 2030.

Other countries’ policy-makers can use the method of this study to assess their achievement of SDGs by the year 2030, evaluate their current trend, make more evidence-based alterations to their health programs if needed, and get as close as possible to attaining sustainable development for all. Future research may focus on finding the contributors to the variations we found among provinces, to help fulfilling SDGs in all areas.

## Conclusion

Iran had favorable declining trends of U5MR and NMR, and has generally achieved SDG 3.2. Nonetheless, there is still inequality among different provinces. Moreover, since the neonatal proportion of U5MR has increased markedly in Iran, special attention needs to be given to NMR determinants. According to our projections, although current decreasing trends are towards NMR improvement, three provincial NMRs will still remain above SDG 3.2 target until 2030. However, if each province operates as the best-performing province in its region, the most suitable outcomes will be attained, and over 90,000 lives will potentially be saved by 2030.

## Supporting information

S1 File Additional information about methodology. (PDF)

S1 Raw data (XLSX)
