# Supplementary material for: Predicting Iran’s achievement to Sustainable Development Goal 3.2: A systematic analysis of neonatal mortality with scenario-based projections to 2030
Source: PLoS One. 2023 Apr 6;18(4):e0283784. doi: 10.1371/journal.pone.0283784 (PMC10079046; doi:10.1371/journal.pone.0283784)
Supplement: S1 File — (PDF) [file pone.0283784.s001.pdf]

Table of Contents

1. Introduction 3

2. Identifying the data sources for neonate and child mortality 4

2.1. Census 4

2.2. Iranian Demographic and Health Survey (IrDHS) 4

2.3. Death registration system 4

3. Statistical analysis for estimating CMR 5

3.1. Analysis of SBH data 5

3.1.1. Maternal Age Cohort (MAC) 5

3.1.2. Maternal Age Period (MAP) 6

3.1.3. Combining two indirect estimates using LOESS regression 7

3.2. Analysis of complete birth history data 7

3.3. Analysis of Death Registration data 8

3.4. Gaussian Process Regression Model 10

3.4.1. Mean function 11

3.4.2. Covariance function 13

3.4.3. Uncertainty with Sampling and Non-sampling Variances 14

3.4.4. Markov Chain Monte Carlo 15

3.5. Spatio-Temporal Model 16

3.6. Model ensemble 18

3.7. Misaligned areal unit in spatio-temporal modeling 18

4. Statistical analysis for estimating NMR 20

5. Figures 22

5.1. Trends of CMR from 1990 to 2017 and NMR from 2010 to 2017 for Iranian provinces . 22

5.2. Projection trends of NMR from 2017 to 2030 for Iranian provinces 37

6. References 46

1. Introduction

We used different data sources and reliable methods to estimate neonatal mortality and child mortality rates in Iran at the national and sub-national levels from the NASBOD project. (1) All available data sources were identified, and comprehensive demographic methods were applied to estimate mortality rates. Due to incompleteness and misclassification of death registration in Iran, this study used information on deaths from all related data sources to analyze mortality data. We employed the Bayesian Model Averaging (BMA) statistical method, with an ensemble of Gaussian Process Regression (GPR) and Spatio-Temporal (ST) models to estimate mortality rates.

GPR can synthesize data from different sources and provide unified time trends and 95% uncertainty intervals for mortality rates at the national and sub-national levels. GPR is usually defined by its mean and covariance function. For this analysis, we considered the Generalized linear mixed model (GLMM) adjusted for spatial and temporal correlations as the prior for mean function and Matern as the covariance function. After defining non-informative prior for hyper-parameters and incorporating empirical data, Markov Chain Monte Carlo (MCMC) method was used to draw samples from posterior distributions of model parameters and predicted mortality rates. GPR also considers both sampling and non-sampling errors in the final uncertainty intervals.

ST model contained three components, spatial, temporal, and spatiotemporal interaction. We used the Besag-York-Mollie (BYM) correlation structure, a conditional autoregressive (CAR) model, to consider the spatial dependency in mortality rates. Non-informative priors were defined for model hyper-parameters, and samples were drawn using the integrated nested Laplace approximations (INLA) method.

2. Identifying the data sources for neonate and child mortality

There were three data sources for estimating the under-five mortality rate in Iran: Death Registration System (DRS), censuses, and Demographic and Health Surveys (DHS). We also used the 12-year (2006-2017) U5MR of DRS. Direct and indirect methods were incorporated into censuses and DHSs. These data sources are explained in detail below. We also used information on neonatal deaths from DRS for 8-year (2010-2017) to estimate NMRs.

2.1. Census

Although there have been many population censuses in Iran in the last decades, the first formal and comprehensive census was performed in 1956. So far, eight censuses have been conducted in Iran. Since 1986, Summary Birth History (SBH) questions have been included in census questionnaires to estimate child mortality indirectly. We used the censuses of 1996, 2006, 2011, and 2016 as indirect data sources to estimate U5MR.

2.2. Iranian Demographic and Health Survey (IrDHS)

DHS is one of the primary data sources for child mortality estimation. DHS collects comparable population-based data on fertility, contraception, maternal and child health, and nutrition in developing countries and is funded by the United States Agency for International Development (USAID). One of the sections in DHS questionnaires is related to the child's health. This section includes questions of SBH and CBH. (2)

2.3. Death registration system

From 1996 to 2001, Research and Technology Deputy, and then (2001 onwards) Health Deputy of the Ministry of Health and Medical Education (MOHME) were responsible for recording death information. National Organization for Civil Registration (NOCR) also registers vital events in

Iran. However, we decided not to use the NOCR database because of delays in the registration of deaths. (3)

3. Statistical analysis for estimating CMR

3.1. Analysis of SBH data

In this stage, we used the methodology developed by IHME for analyzing SBH data. In this method, SBH data are analyzed by two different approaches: cohort and period. (4)

3.1.1. Maternal Age Cohort (MAC)

Maternal Age Cohort (MAC) method is a modification of WHO version in two ways. (5) One is that it directly estimates the under-five mortality rate, and the second is that the country's random effect is incorporated into the model. This method is based on two regression equations. In this method, under-five mortality and reference time equations are estimated using summary birth history data with the mother's age as a proxy for children. Then, by calculating the antilog of the logit of the mortality rate, it is converted into the under-five mortality rate.

Component of reference time is:

$$refrence\ time_{ijk} = \beta_{0i} + \beta_{1i}\frac{CD_{ijk}}{CEB_{ijk}} + \beta_{2i}CEB_{ijk} + \beta_{3i}\frac{P(15-19)_{jk}}{P(20-24)_{jk}} + \beta_{4i}\frac{P(20-24)_{jk}}{P(25-29)_{jk}} + \varepsilon_{ijk}$$

The logit component of under-five mortality rate is:

$$logit(5q0_{ijk}) = \beta_{0i} + U_{ij} + \beta_{1i}logit(\frac{CD_{ijk}}{CEB_{ijk}}) + \beta_{2i}CEB_{ijk} + \beta_{3i}\frac{P(15-19)_{jk}}{P(20-24)_{jk}} + \beta_{4i}\frac{P(20-24)_{jk}}{P(25-29)_{jk}} + \varepsilon_{ijk}$$

Subscripts in two equations are as follow:

$i = 5 - year\ maternal\ age\ group\ \in \{15-19, 20-24, \dots, 45-49\}$

$j = country$

$k = survey$

$P(\dots) = parity\ (average\ CEB) for\ specified\ maternal\ age\ group$

$CD_i = total\ dead\ children\ from\ maternal\ age\ group\ i$

$CEB_i = total\ children\ ever\ born\ from\ maternal\ age\ group\ i$

For each age group of mothers, one U5MR and one reference time are calculated.

3.1.2. Maternal Age Period (MAP)

In the second approach, Maternal Age Period (MAP) method finds the distribution of child birthdays and death days for different categories of mothers, stratified by region, age, and the number of children ever born/dead children. The expected numbers of children ever born (CEB) and children dead (CD) were calculated using these distributions for every year before the survey. In the next stage, we added the numbers of CEB and CD for all strata and every year. Finally, we calculated the ratio of CD to CEB and found the under-five mortality. This method has one regression equation. Below is the equation of the MAP method:

$$logit(5\text{q}0_{tjk}) = \beta_t^0 + U_{tj} + \beta_t^1 logit(\frac{CD_{tjk}}{CEB_{tjk}}) + \varepsilon_{tjk}$$

In this method, the subscripts are:

$t = index\ of\ calender\ time\ \in\ [0,24]$

$j = country$

$k = survey$

$CD_{tjk} = total\ dead\ children\ in\ time\ bin\ t$

$CEB_{tjk} = total\ children\ ever\ born\ in\ time\ bin\ t$

For every year before the survey, one U5MR is obtained. (4)

3.1.3. Combining two indirect estimates using LOESS regression

We smoothed and combined two estimates obtained from MAC and MAP by using Loess. Loess has the advantage of not limiting the estimates to a linear trend over time. Here, we excluded the U5MR obtained from the 15-19 years old mothers because MAC estimates of this age group are overestimated. (4)

3.2. Analysis of complete birth history data

At first, to analyze CMR using CBH, we converted the date of birth, date of death, and age of death of participants into Century Month Code (CMC) format.

CMC=12 (year of birth - 1900) + month

Then, we determined and counted the deaths of under-five years (in CMC format) in the year of study. Here, if the age at death was under 60 months, and the date of death in CMC format located within the year of study, we have a relevant death event.

In the next step, to calculate the time of exposure to the risk of dying for a person aged under-five year and in a specific year, we determined what percentage of that specific year is at risk of dying for one person under 60 months. In total, we considered the following scenarios. If individual aged older than 60 months of the date of birth (in CMC format) was out of the specific year, the time of

exposure for these persons was considered by 0. On the other hand, for a specific year, if an individual aged under 60 months, we determined what percentage of that year person is survived and at risk of dying. For example, if the individual for the whole year were under 60 months and survived, the time of exposure would be 1, and if for six months were under 60 months and survived, the time would be 0.5. Then, the number of death events and exposure time were multiplied by sampling weight, which was provided in the DHS study. Next, the number of death and time of exposure were summed. Finally, we divided the cumulated death events by the sum of exposure times and multiplied by 1000. (6)

3.3. Analysis of Death Registration data

A common problem related to analyzing DRS data is missing values of age and sex. To estimate U5MRs, subjects with missing age values will drop out of the numerator of calculated rates. We have used Amelia II package (R 3.5.3) to impute missing values of age and sex variables. Amelia implements a bootstrapping-based algorithm to perform multiple imputations, and it includes useful diagnostics for imputed values. (7)

Besides the problem of missing values, the death registration system's incompleteness leads to underestimating mortality rates. Therefore, we should adjust for the degree of incompleteness in the analysis. We estimated DRS's completeness using a model that compares the level of U5MRs from DRS with the other data sources. (8) In the first step, we apply a sub-national level regression of the logarithm of mortality rates on year and an indicator variable adjusting for the incompleteness in the level of DRS mortality rates:

$$log_{10}5q0 = \alpha + \beta_1.t + \beta_2.I_{DRS} + \varepsilon_t$$

Where t is time (year), I is indicator variable for mortality rates that are obtained from DRS and ε_t is the error term. The magnitude of the coefficient for this indicator shows the completeness of DRS data. If the coefficient of the indicator variable is statistically significant at level 0.05, we conclude that DRS has incompleteness for that province. As is shown in the equation, this model allows completeness to vary over time. Completeness and its standard error estimated in this stage will be used in GPR model, which is explained in the following section.

3.4. Gaussian Process Regression Model

Gaussian Process is a generalization of a Gaussian (normal) probability distribution, which extends any finite linear combination of random variables to functions. (9) Like multivariate normal distribution, GP has a mean and covariance with the difference that these mean and covariance can be functions. A GP is entirely described by its mean and covariance function. These two functions are determined by data information and properties. For this reason, the Bayesian framework is applied for improving the model to conclude accurate and more reliable estimates. Since our target values are mortality rates over time, we used Gaussian Process Regression to draw an inference based on these values. (10) We intend to combine time series of different data sources for a unique estimate of U5MRs with a 95% uncertainty interval.

We considered the logarithm of under-five mortality rates to have a normal distribution. The mean is a function of year and an indicator variable that shows whether mortality rates are from DRS. The variance of mortality rates includes both sampling and non-sampling errors described in the following parts. Since in some provinces DRS undercount child deaths, the bias term (β_{DRS}) is added to mortality rates to adjust for incompleteness. If the indicator coefficient for a province was significant, we set a normal prior for the incompleteness coefficients with the mean equal to the DRS indicator's coefficients and the variance equal to the variance of the coefficients estimated in this equation.

$$\log_{10}(5q0_{t,s}) \sim N(\mu_{t,s}, \sigma_{t,s}^2 + \omega_s^2)$$
$$\mu_{t,s} = f(t) + \beta_{DRS}.I_{s=DRS}$$
$$f \sim GP(M, C)$$
$$\beta_{DRS} \sim Normal(\mu_{DRS}, \sigma_{DRS}^2)$$

Where: t is time (year); s is source type; f is a function that forms GP.

f describes GP with a mean and covariance function. Mean function M is Generalized Linear Mixed Models (GLMM) adjusted for spatial and temporal correlations, and covariance function C is a Matern function described in the following sections.

3.4.1. Mean function

For the mean function, we used a GLM model adjusted for correlation between neighboring provinces and nearby time points. This model uses this information to predict for province-years where no data or sparse data is available. We have used the additional information of covariates (including wealth index, years of schooling, and urbanization)[1] to make this prediction more accurate. The model specification is defined in the following equation:

$$log(5q0)_{it} = \beta_0 + \beta_1 WI_{it} + \beta_2 YOS_{it} + \beta_3 Urban_{it} + b_{0i} + b_{1i} year_i + \varepsilon_{it}$$

Where:

1. WI, YOS, and Urban is abbreviations for the mean wealth index, mean years of schooling, and percent of urbanization in each province-year.

2. b_{0i} is the random province effect and allows each province to have had the mean of CMR which, was different from other provinces. b_{0i} has a Normal (0, σ_{b0}^2) prior.

1. b_{1i} term allows changes in log $(5q0)$ over time in each province to be different from other provinces. b_{1i} has a Normal (0, σ_{b1}^2) prior.

[1] The wealth index is obtained as a composite measure of a household's cumulative living standard. We used data on household's ownership of some selected assets (e.g. television and car), materials used for housing construction, and having some facilities (e.g. water access type). These data are gathered annually by Statistical Center of Iran using standard questionnaire. Totally, we have used 24 asset indicators for each family as input data in a Principle Components Analysis (PCA). We extracted the first component of PCA which explained 22.4% of all variances as the wealth index. We aggregated the family level data using appropriate weights to obtain the average wealth index by province-year.

2. ε_{it}, the error term that has a Normal $(0,\sigma^2)$ prior.

Then, to calculate changes of dependent variable across time and space, we applied an additional regression analysis. In this order, we estimated the residual (predicted - observed dependent variable) of GLMM for each data point and then ran a local regression in two dimensions on the residual. Considering that the residuals contain valuable information that varies systematically across place and time but cannot be directly observed, this process allows us to predict how much the observed dependent variable varies from the mixed-effects model's prediction and account for these differences. Local regressions were done by cycling through every observation in the dataset, weighting each observation in the dataset relative to it, and finding the residual s's weighted mean. We estimated adjacency matrices over province and year. Then by using a weighting scheme similar to the tricubic weights used in traditional LOESS local regression, we weighted all observations j relative to observation i in time:

$$W_{T_{ij}} = \ (1 - (\frac{|year_i - year_j|}{ArgMax(|year_i - year_j| + 1)})^{\lambda})^3$$

Where $\lambda = 2$ is a parameter that can be adopted to raise or decline the level of smoothing across time. We calculated the weight matrix for adjacent provinces whose elements for provinces that are neighbors equal 1 and 0 otherwise. Once these weights have been calculated, weighting every observation in the data set relative to the one being predicted, it a simple matter of calculating a weighted average of the residuals from regression. This "predicted residual" is then added back onto the prediction, creating an estimate that more closely considers aspects of the data that cannot be captured by a simple covariate model. (11)

3.4.2. Covariance function

The covariance function in GP determines how each data point can borrow information from neighboring points. Finding a suitable covariance function of GPR is a critical part of the analysis because it adjusts data variation by its parameters. Among the various covariance functions for GP, Matern function was chosen because it is a suitable one for time series data, and its parameters entirely define it. Matern defined for two points separated by a distance d as:

$$C(d) = \lambda^2 \frac{1}{\Gamma(\upsilon)}\left(2\sqrt{\upsilon}\,\frac{d}{\rho}\right)^{\upsilon} K_{\upsilon}\left(2\sqrt{\upsilon}\,\frac{d}{\rho}\right)$$

Where: d is time here;

Γ is gamma function;

K_{υ} is modified Bessel function of the second kind

$$\lambda = \sqrt{\frac{1}{T}\sum_{i=1}^{T}(\log_{10} 5q0_t - M(t))^2}$$

$$\rho \sim Uniform(10, 40)$$

$$\upsilon = \begin{cases} 0.8 & s = DRS \\ 2 & otherwise \end{cases}$$

The calculated values from C (d) are placed in a symmetric matrix of prediction years. This function includes three parameters: one is called "amplitude," which calculates the deviation between under-five mortality rates and GP mean function over time; another is the "scale" parameter, which captures the correlation between years. This parameter is defined by uniform distribution and updated by Bayesian inferences. The third parameter, "degree of differentiability,"

controls samples' smoothness from the Gaussian Process. Mortality rates obtained from DHS and censuses have a disagreement in reporting values and need to be made smoother. We assume less degree of smoothness for death registration observations and a greater degree of smoothness for observation from other mortality sources. We set the same values that IHME used to maintain comparability.

3.4.3. Uncertainty with Sampling and Non-sampling Variances

According to existing different data sources for estimating child mortality rates and considering sampling variance, we are interested in estimating the disagreement between data sources in terms of uncertainty. A critical output from GPR is an estimate of both the expected value of mortality rates and their uncertainty. The uncertainty estimate captures both uncertainties in prior mean function and the data variance from each observation. Data variance is a function of both sampling and non-sampling error.

We calculate sampling variance of rates assuming a binomial distribution, with sample size equal to the number of children between birth and exact age five depending on the data source and probability equal to the under-five mortality rate. We choose the size of the birth cohort for DRS data points. The variance that is obtained by using this distribution is the sampling error mortality rate. According to delta method, the variance of this distribution is given by

$$Var\left(log_{10}(5q0)\right) = \sigma_{t,s}^2 = \frac{1}{(5q0_{t,s}\log(10))^2}\,\frac{5q0_{t,s}(1-5q0_{t,s})}{N_t}$$

Non-sampling variance captures the variation between data sources. Unlike the sampling variance, we specify a prior distribution for it. The more variation in data sources, the higher the variance parameter and, therefore, the more uncertain the predictions.

$$\frac{1}{\omega_s} \sim Gamma\ (0.1*\tau_s\ ,\ 0.1)$$

$$\frac{1}{\tau_s} = MAD\,[\log_{10}(5q0_{t,s}) - f(t)]^2 - \frac{1}{T}\sum_{t=1}^{T}\sigma_{t,s}^2 - \delta_{s=DRS} * \frac{1}{T}\sum_{t=1}^{T}\sigma_{DRS}^2$$

We have three source types in our study: census, Demographic & Health Survey, and Death Registration System. We excluded the variance of DRS' incompleteness in high-quality provinces. The MAD is median absolute deviation, which has a good performance in the presence of outliers. Gamma distribution is suitable for estimating variance in Bayesian inferences. The sum of these two variances (sampling and non-sampling) result uncertainty interval.

3.4.4. Markov Chain Monte Carlo

We used Bayesian inference to update predicted mortality rates as a posterior in Bayes rule by combining data and prior probability distribution over parameters in mean, covariance function, and the regression model. These predicted series are updated by the data within each province using GPR model. We employed Markov Chain Monte Carlo (MCMC) to sample from posterior probability distribution, describing the probability of a true model given the under-five mortality rates that we observe in a province. Except in special circumstances, the posterior distribution for model parameters cannot be calculated analytically and must be approximated. Using MCMC, we draw samples from the model's posterior distribution to estimate the median and 95% uncertainty intervals of the mortality rate across provinces over the years. We draw 12000 samples for every province after discarding the first 6000 burn-in samples. Across all samples for a given province-year combination, we used median values, 2·5 and 97·5 percentiles of each outcome as the point estimate and upper and lower confidence intervals.

To do MCMC calculations, we used RStan package version 2.19.2 in R software. RStan can employ complex new statistical methods (such as Hamiltonian Monte Carlo) in MCMC. It has a similar syntax to BUGS language for Bayesian analysis and compiles C++ codes based on user-written scripts. By using RStan package, we can apply complex models to the data. It is also an open-source package that is easily accessible through the internet. We employed GPR method, which was developed by GBD study and all the parameters were the same compared to the methodology for under-five mortality in GBD 2015.

3.5. Spatio-Temporal Model

In this study, we employed the spatiotemporal model in a Bayesian framework to estimate CMRs in all provinces of Iran. To take the spatial dependency into account, we use Besag-York-Mollie (BYM) correlation structure under conditional autoregressive (CAR) models.

We describe the model as follows:

$$log(5q0)_{it} = \beta_0 + \beta_1 WI_{it} + \beta_2 YOS_{it} + \beta_3 Urban_{it} + u_i + \upsilon_i + \gamma_t + \varphi_t + \gamma_{it} + \varepsilon_{it}$$

Where:

1. u_i is the spatial unstructured random effect in province i and has a Normal (0, σ_u^2) prior;

2. υ_i is the spatial structured random effect in province i and has a conditional autoregressive prior as follows;

$$\upsilon_i | \upsilon_{-i} \sim Normal\,(\frac{1}{\mathcal{N}_i} \sum_{j=1}^{n} a_{ij}\, \upsilon_j, s_i^2)$$

$$a_{ij} = \begin{cases} 1 & If\ i\ and\ j\ are\ neighbours \\ 0 & Other\ wise \end{cases}$$

$$s_i^2 = \frac{\sigma_v^2}{\mathcal{N}_i}$$

As is shown in the formula, spatial random effects for each province have a normal distribution, with its mean equal to the weighted average of spatial random effects of neighboring provinces and a variance that decreases as the number of neighboring provinces increases. This neighborhood structure usually is defined as a weight matrix whose elements equal 1 for provinces that are neighbors and 0 otherwise.

3. γ_t is the temporal unstructured random effect in year t and has a Normal (0, σ_t^2) prior;

4. φ_t is the temporal structured random effect in year t and has a random walk of order-2 prior as follows;

$$\varphi_t \mid \varphi_{-t} \sim Normal(\varphi_{t+1}, \tau_\varphi) \quad for\ t = 1$$
$$\varphi_t \mid \varphi_{-t} \sim Normal(\frac{\varphi_{t-1} + \varphi_{t+1}}{2}, \frac{\tau_\varphi}{2}) \quad for\ t = 2, ..., T-1$$
$$\varphi_t \mid \varphi_{-t} \sim Normal(\varphi_{t-1}, \tau_\varphi) \quad for\ t = T$$

5. γ_{it} is interaction term between spatially and temporally structured random effects. We assume the distribution of the interaction effect as:

$$\gamma \sim Normal\ (0, \sigma_\gamma^2 (R_v \otimes R_\varphi)^{-1})$$

Where R_v and R_φ are the adjacency and random walk (RW2) dependency matrices, respectively.

We have used non-informative priors for model hyper-parameters such as variance components and fitted this model using RINLA package version 20.03.17 from R software. (12)

3.6. Model ensemble

As described in the main text, we developed an ensemble of GPR and ST models to estimate CMRs. We applied a BMA method to combine these two models and generate final estimates of CMRs. The BMA method combines posterior distributions of each individual ensemble model to provide final estimates. The share of each model in the final estimates determined the basis of out-of-sample performance. (13) Each model was weighted based on a monotonically declining function as follows:

$$W_i = \frac{\psi^{(N-rank_i)}}{\sum_{j=1}^{N}(\psi^{(N-j)})}$$

Where N is the number of models and ψ is a parameter influencing the relative weighting of models. We set $\psi = 1.2$ based on previous studies. (11)

3.7. Misaligned areal unit in spatio-temporal modeling

Increasing the number of provinces during this period (24 provinces in 1990 versus 31 provinces in 2017) was a challenging issue in modeling mortality data. This increase leads to changes in the neighborhood structure of provinces in the Spatio-temporal model. Nonetheless, many commonly-used Spatio-temporal models work with a unique neighborhood matrix throughout the years. One solution for this problem is to aggregate the newly added provinces based on the country's old administrative divisions. This solution is usually known as backward alignment. Since we had access to district codes for many data sources, the misalignment problem can be solved for most provinces. We extracted the district codes based on the Statistical Center of Iran's administrative

division in 2011 and then attempted to form the new provinces by aggregating related districts in the preceding years. Unfortunately, for some data sources, including DHS 2000 and the census 1996, the data had been collected only at the provincial level, and there was no information available at the district level. For these data sources, we have used a statistical model that uses U5MRs of old provinces to predict U5MRs for newly added provinces. Imagine province A is subdivided into two provinces, B and C. For provinces with access to the recent administrative division (B and C), we used a weighted population-based average of U5MRs to estimate mortality rates based on the old administrative division (A). For data sources that we had access only to the old administrative division (A), we added ID codes for new provinces (B and C) with missing values for U5MRs. For each misaligned areal units, we put all related provinces (A, B, and C) from all sources together in a data set and fitted the following model:

$$log(5q0)_{ijt} = \alpha_i + \gamma_j + \beta_1 WI_{it} + \beta_2 YOS_{it} + \beta_3 Urban_{it} + \beta_4 year$$

Where α_i is the fixed effects of provinces (that could be A, B, and C) and γ_j is the fixed effects of data sources (three SBH and one CBH). Using this model, we can predict the mortality rates for misaligned provinces of DHS 2000 and the census 1996 according to the recent administrative division of the country.

4. Statistical analysis for estimating NMR

The NMR was modeled as the product of two components: the expected NMR and a province-year-specific multiplier. The expected NMR provides the expected association between the NMR and the current level of U5MR in a particular province and year. We searched for parametric and non-parametric patterns of the global association between the expected NMR and U5MR, and we found that a quadratic function well captures the association between the log (NMR) and log (U5MR) (figure1). Previous studies investigated the relation between the ratio (log (NMR)/log (U5MR) – log (NMR)) and estimated U5MR at the global level. (14) We compared the relation between ratio and estimated U5MR in our data with other studies and found a similar relation. (figure2).

The second component, the province-year-specific multiplier, allows provinces to have NMRs that are higher or lower than expected given the current U5MR, compared with the global association. The province-specific multipliers were modeled with GPR and ST models. We considered the quadratic form of CMR estimates as explanatory variables in GPR's mean function and ST model to estimate NMR.

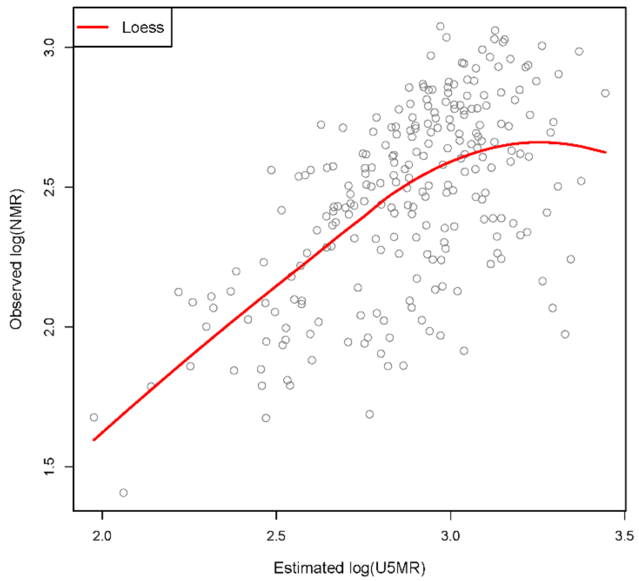

Figure1: Observations log(NMR) are displayed with grey dots and plotted against log(U5MR). The loess fit to the observations is shown in red line.

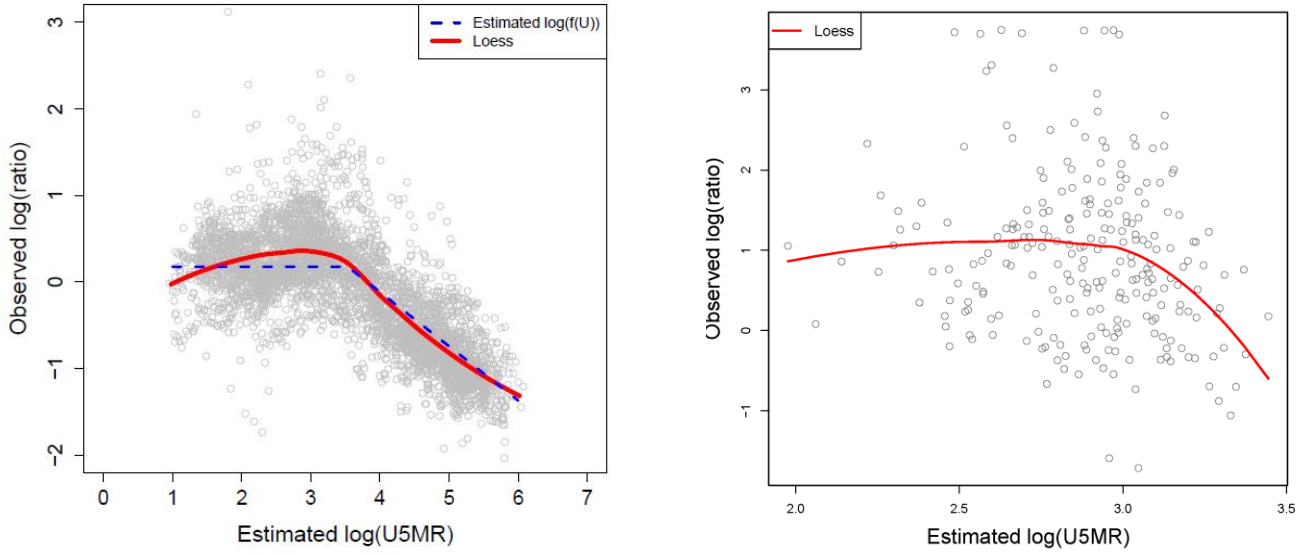

Figure2: The left plot shows the relation between the observed log(ratio) and predicted log(U5MR) at the global level (14), and the right plot shows this relation between Iran's mortality data.

5. Figures

5.1. Trends of CMR from 1990 to 2017 and NMR from 2010 to 2017 for Iranian provinces

Markazi

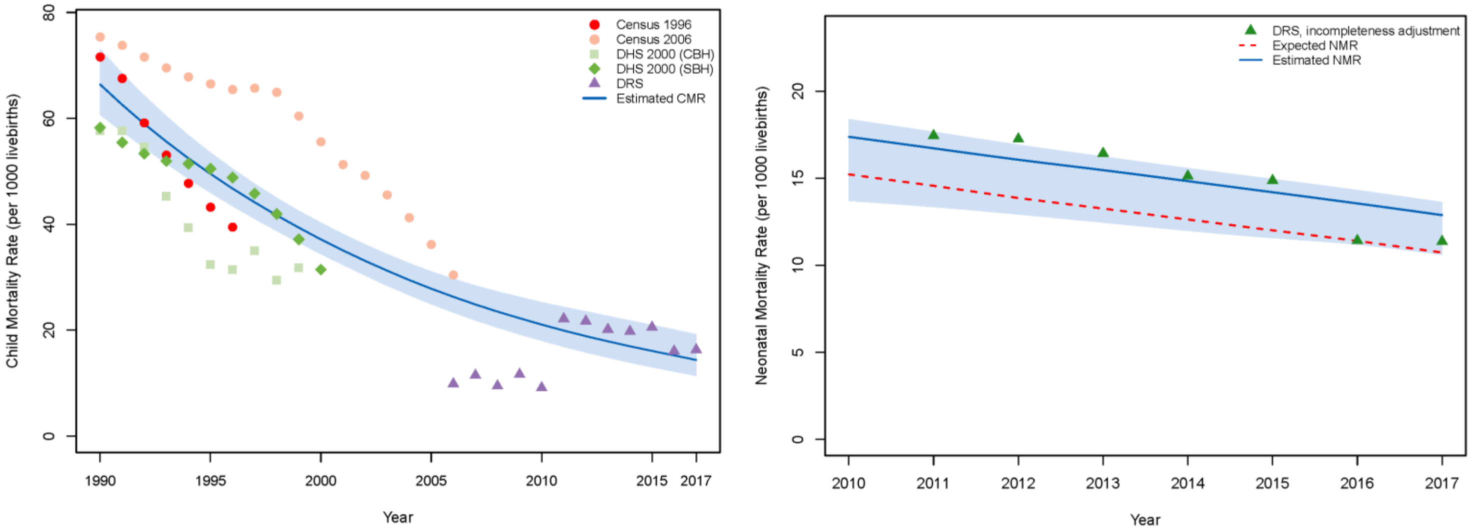

Gilan

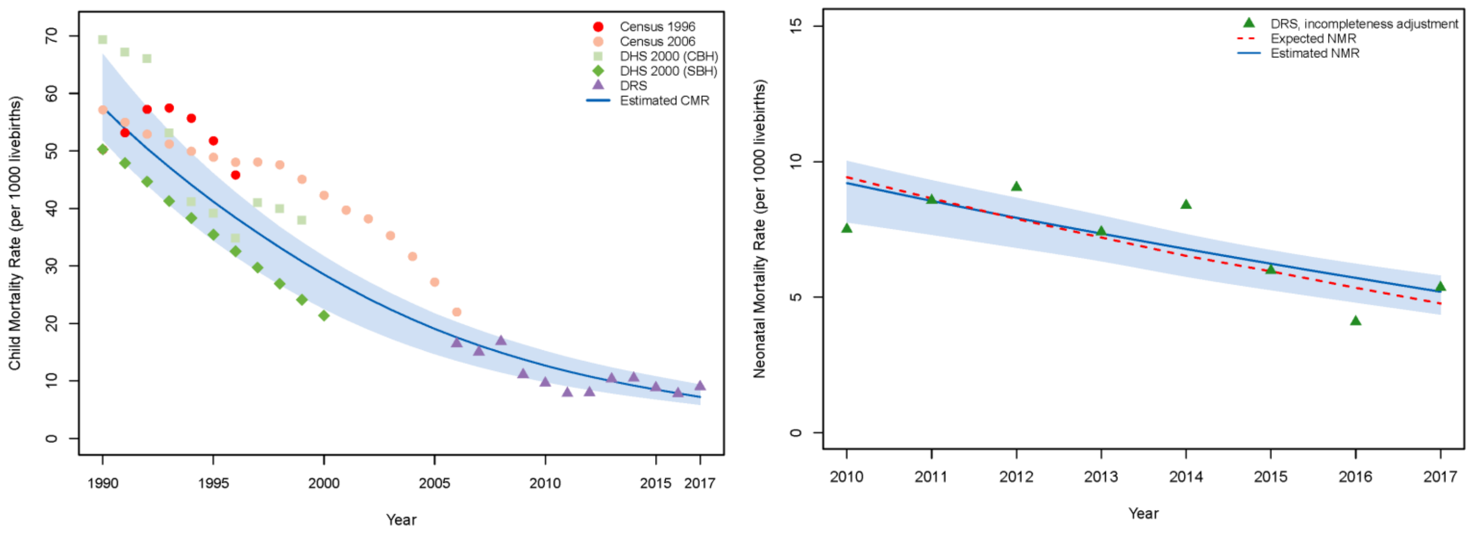

Mazandaran

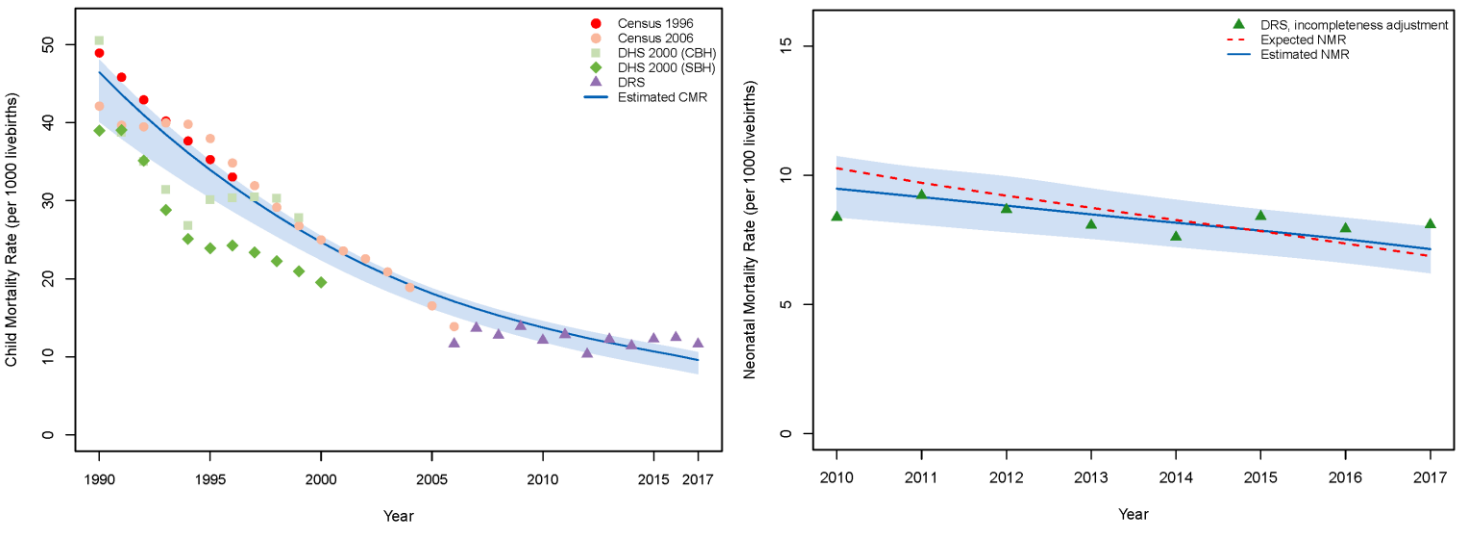

Azerbaijan, East

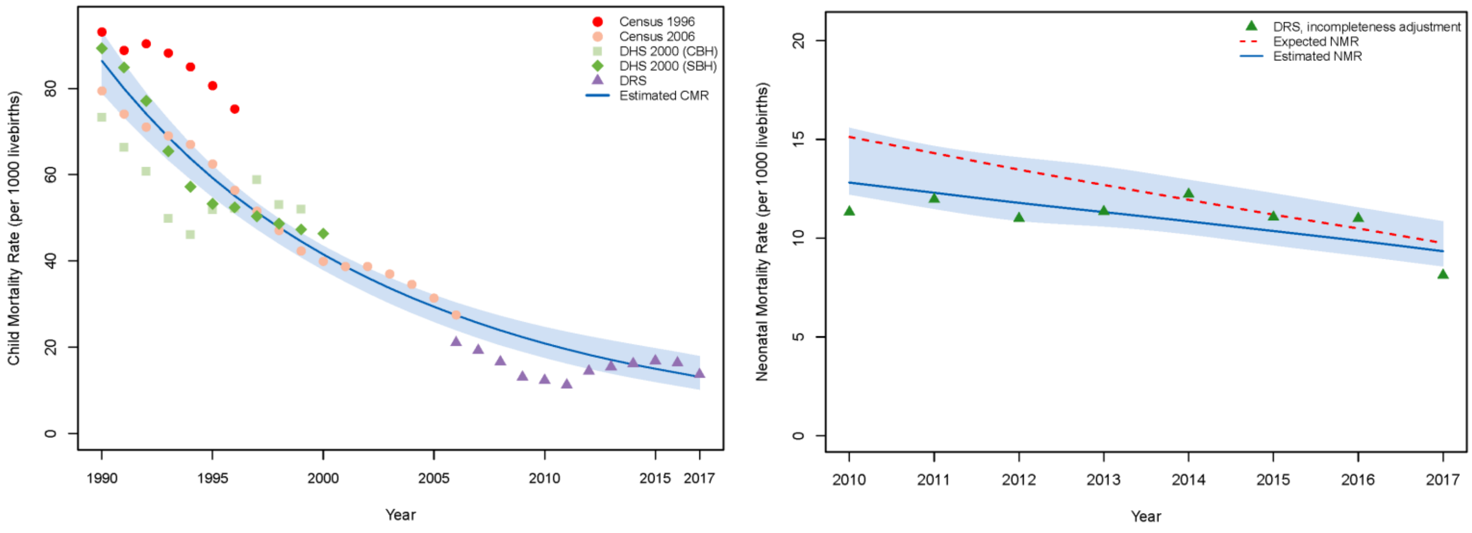

Azerbaijan, West

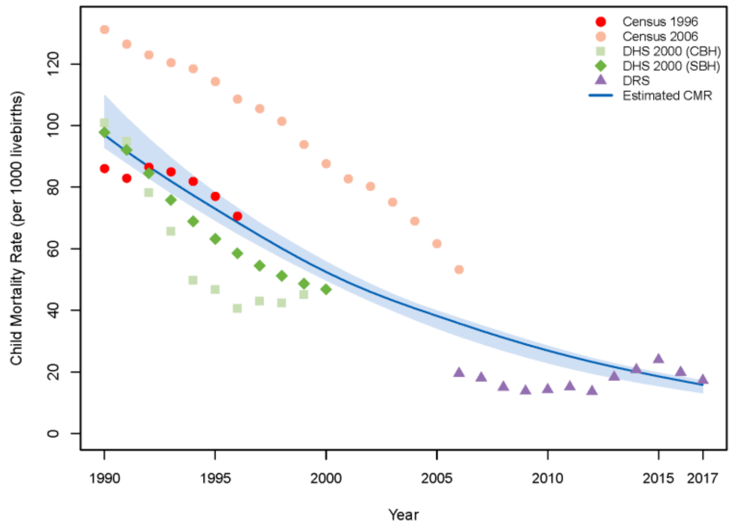

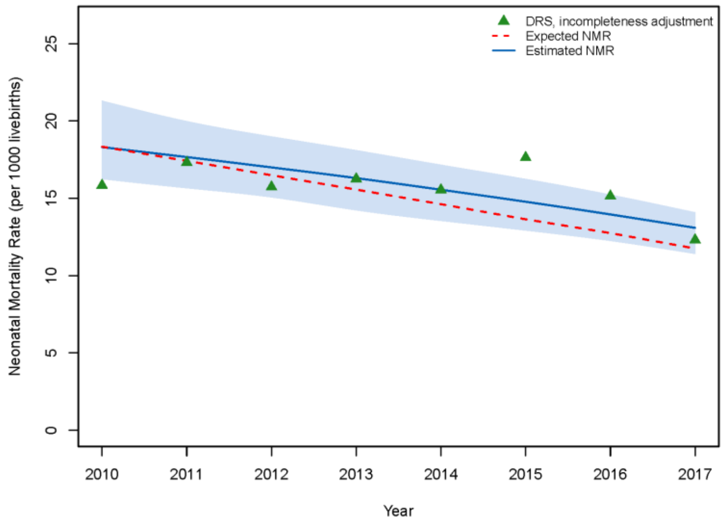

Kermanshah

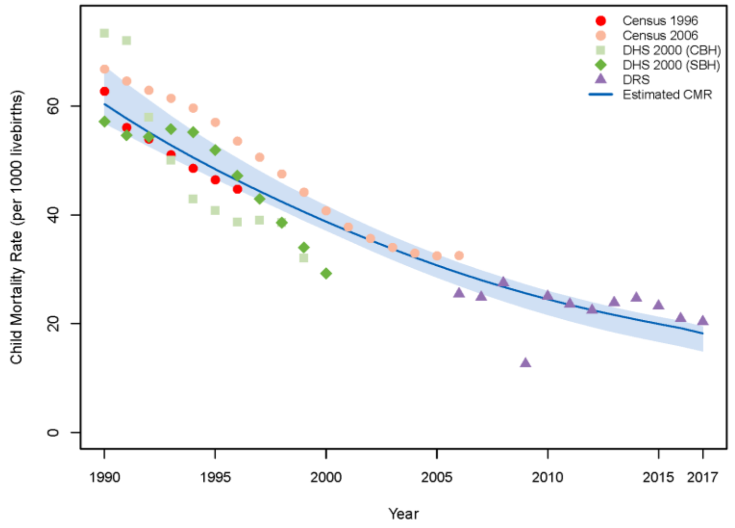

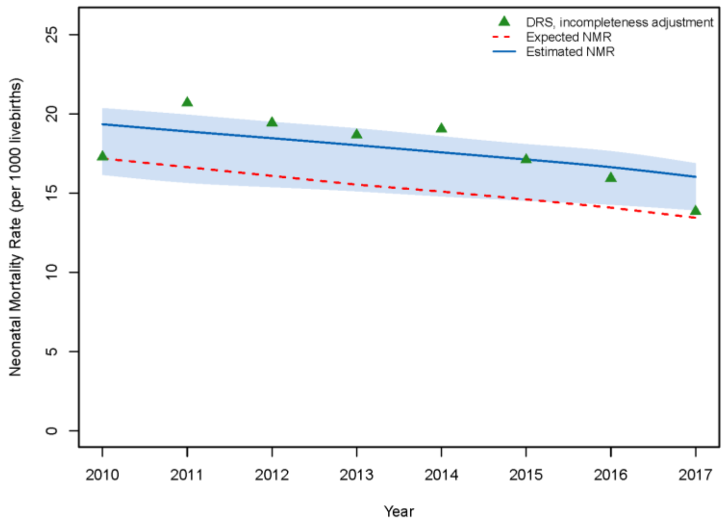

Khuzestan

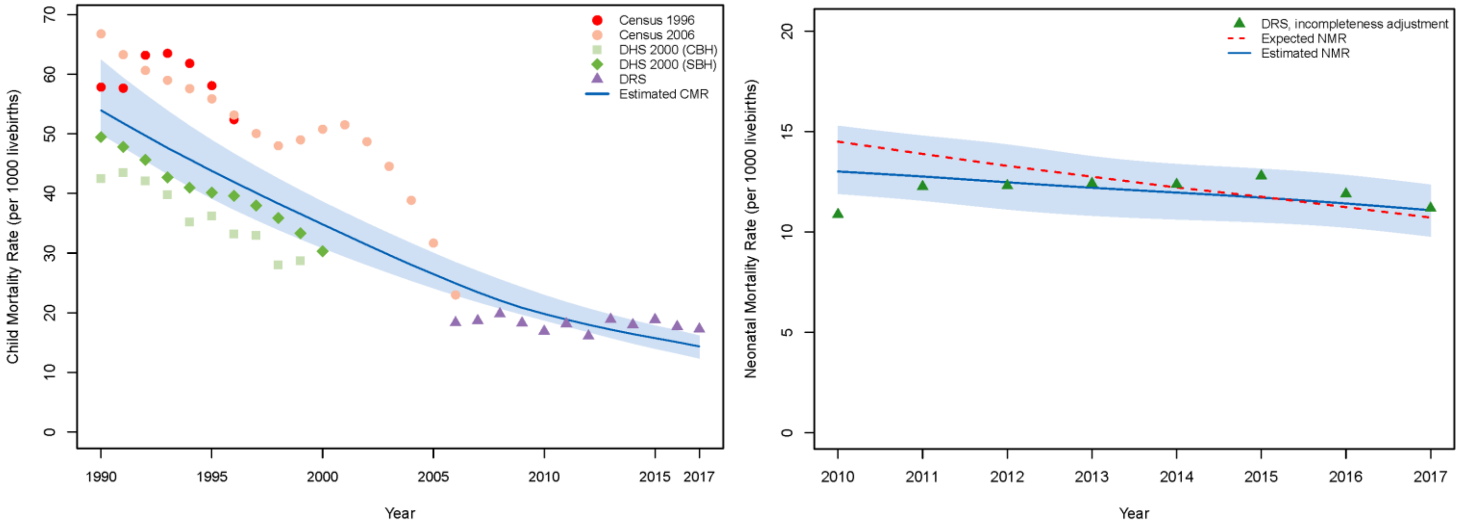

Fars

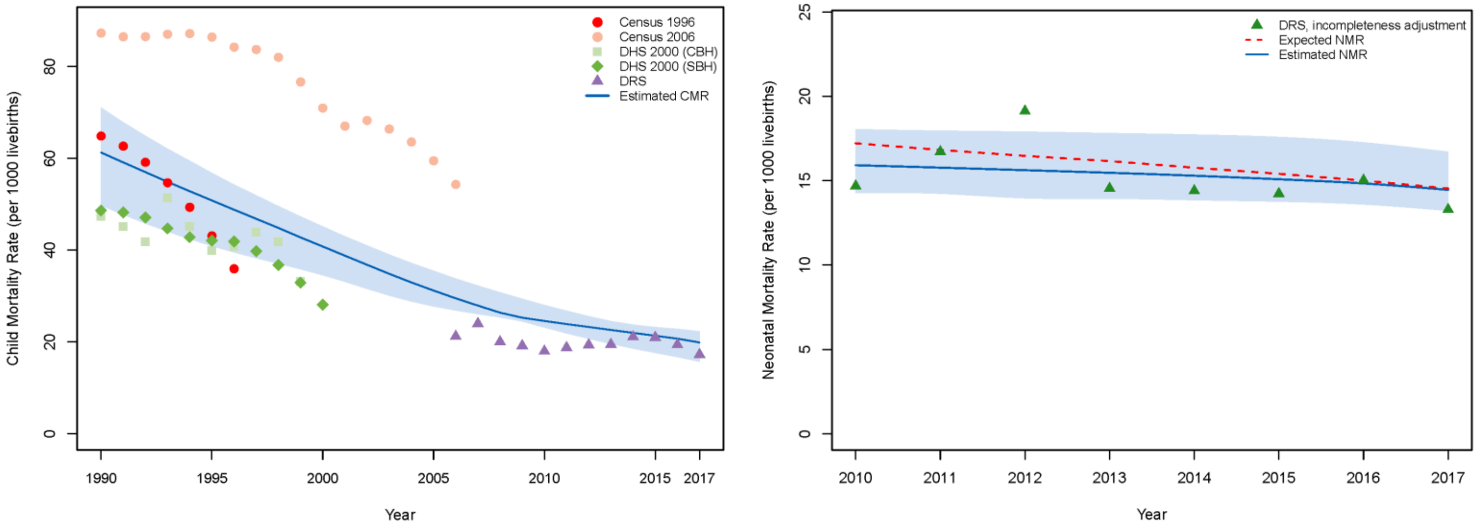

Kerman

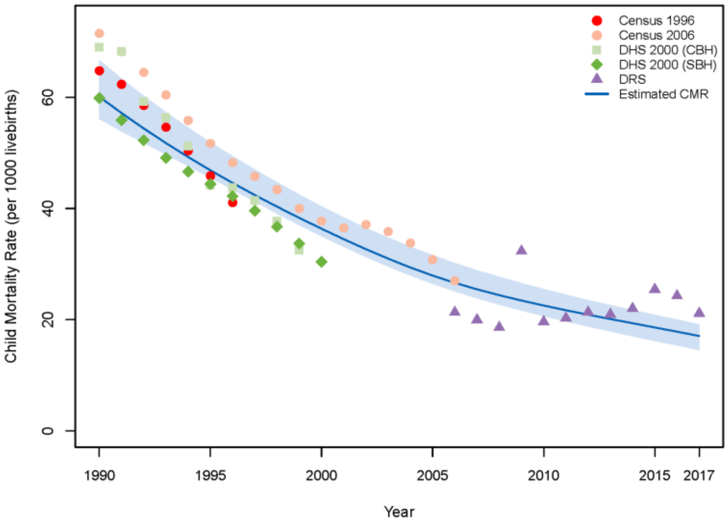

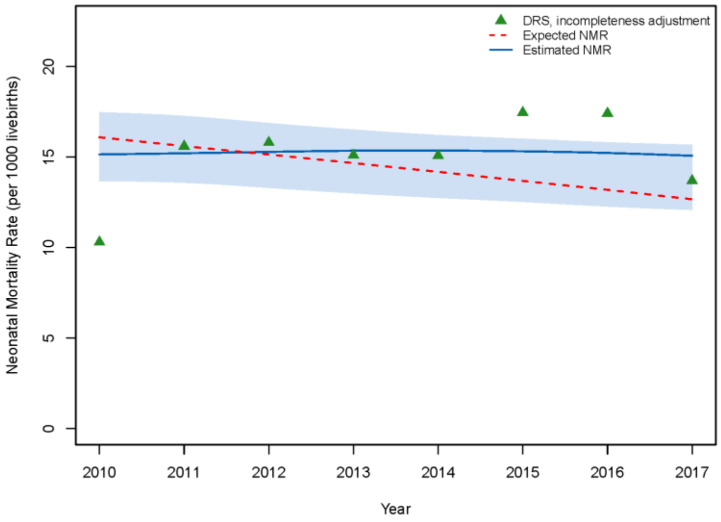

Khorasan, Razavi

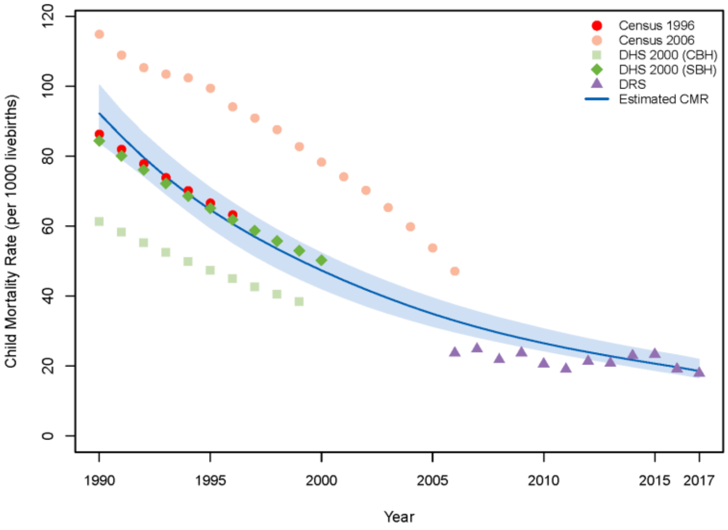

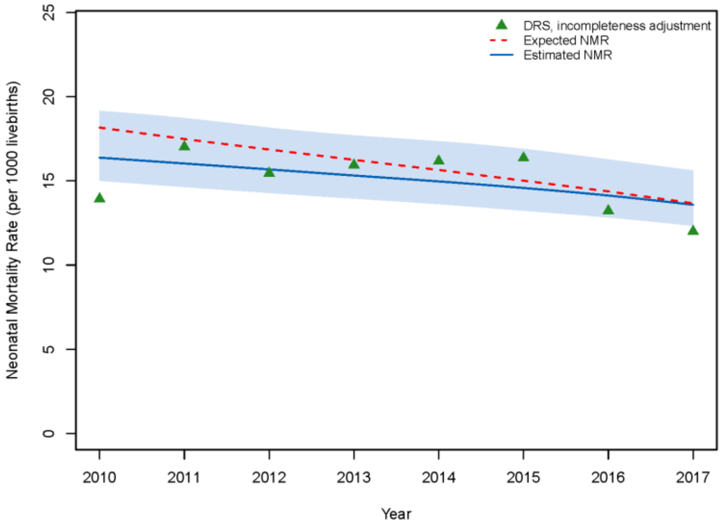

Isfahan

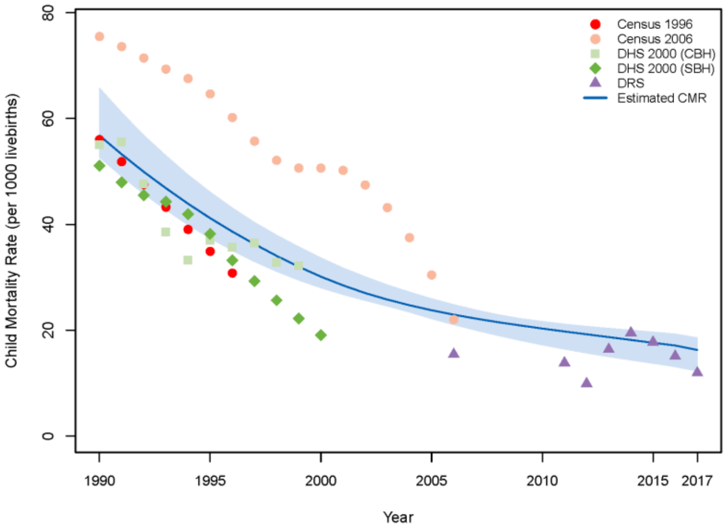

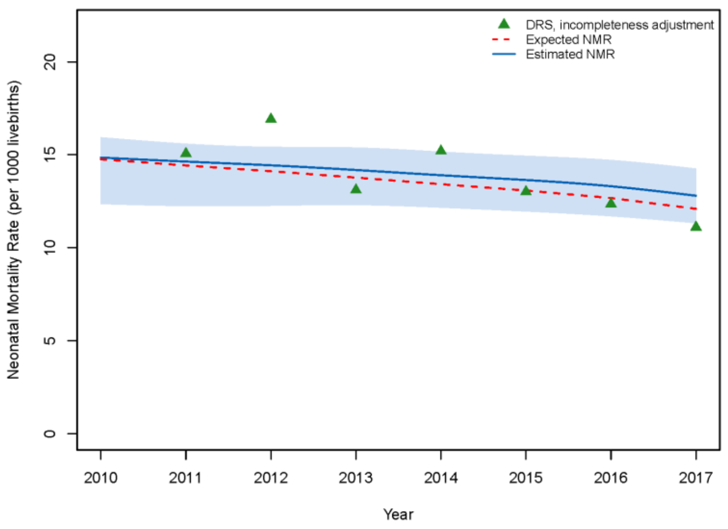

Sistan and Baluchistan

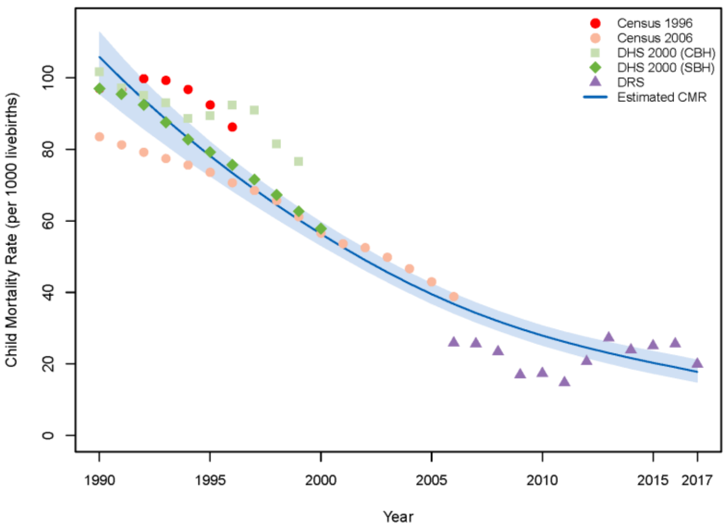

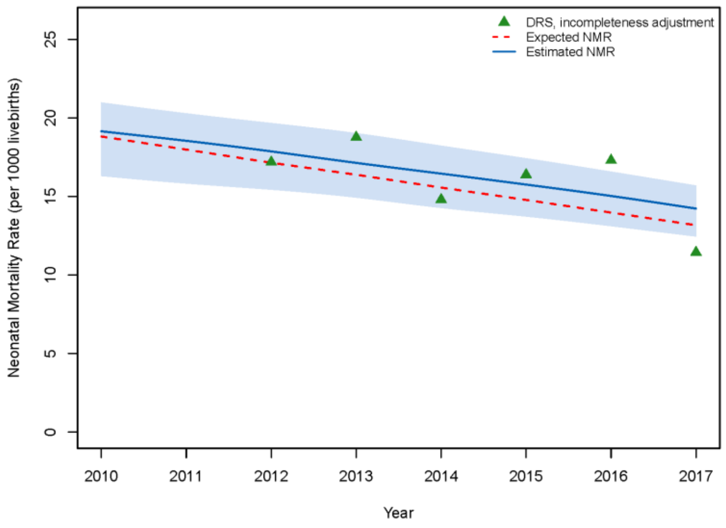

Kordestan

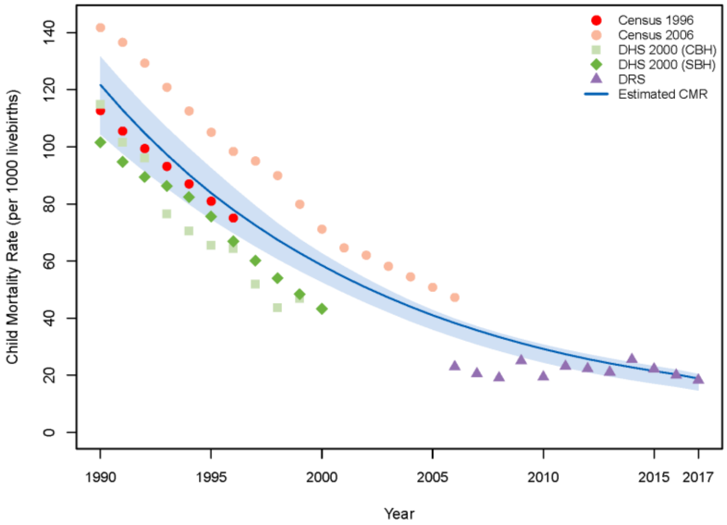

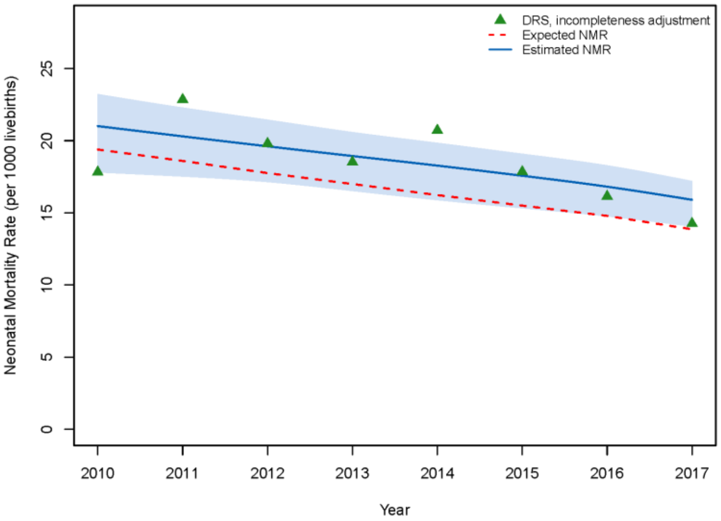

Hamadan

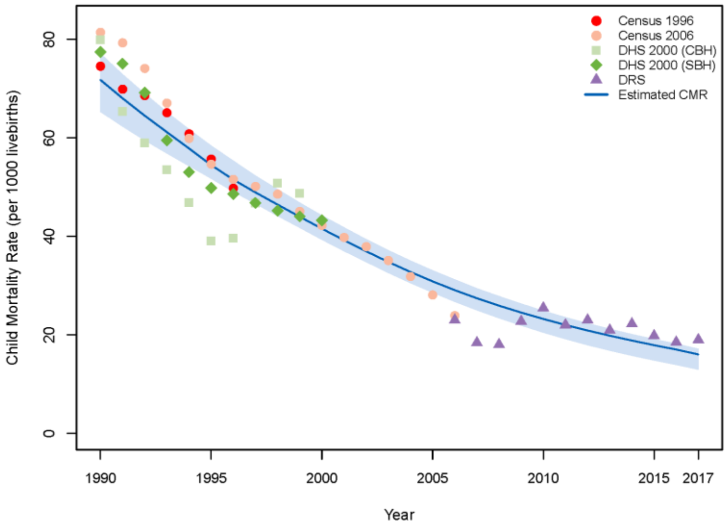

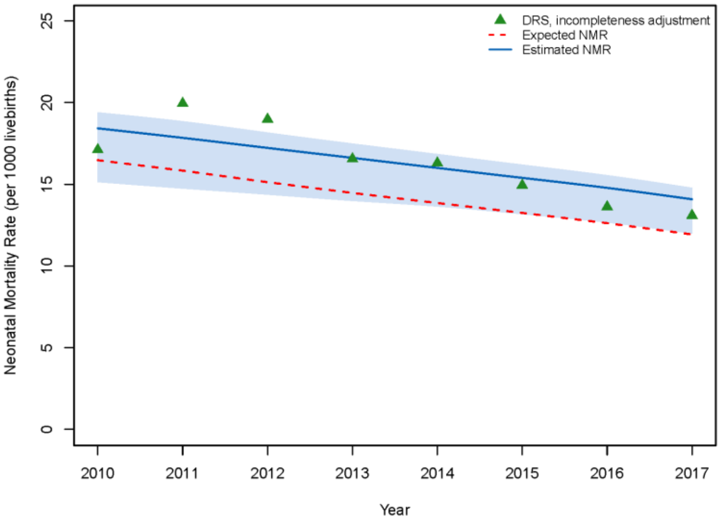

Chahar Mahaal and Bakhtiari

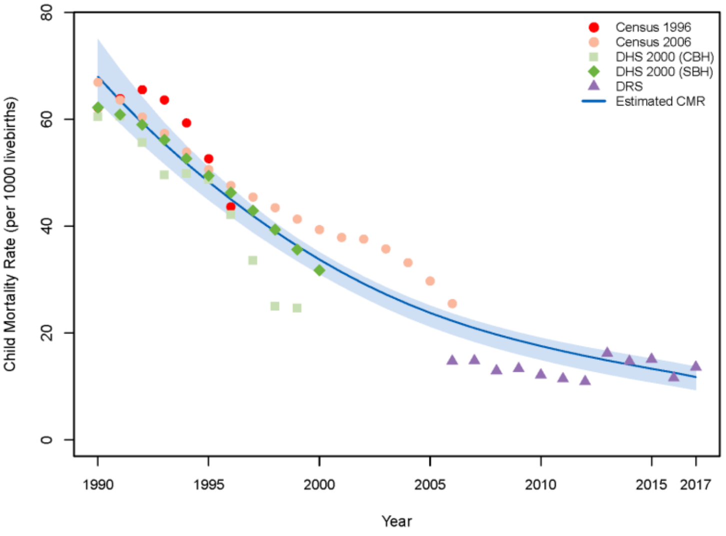

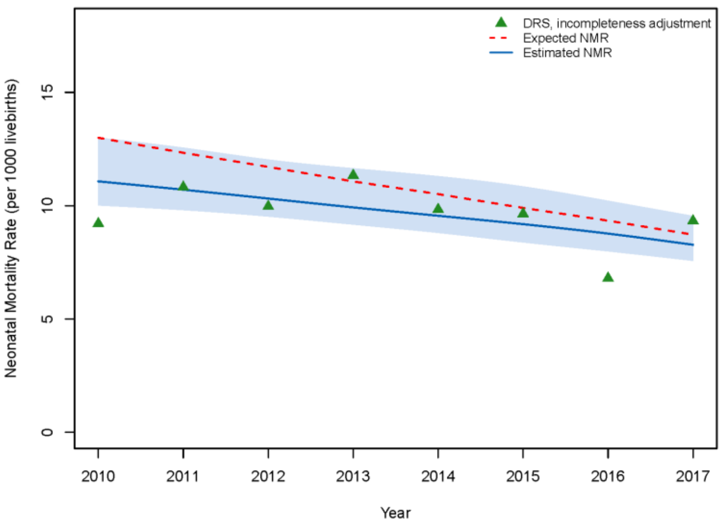

Lorestan

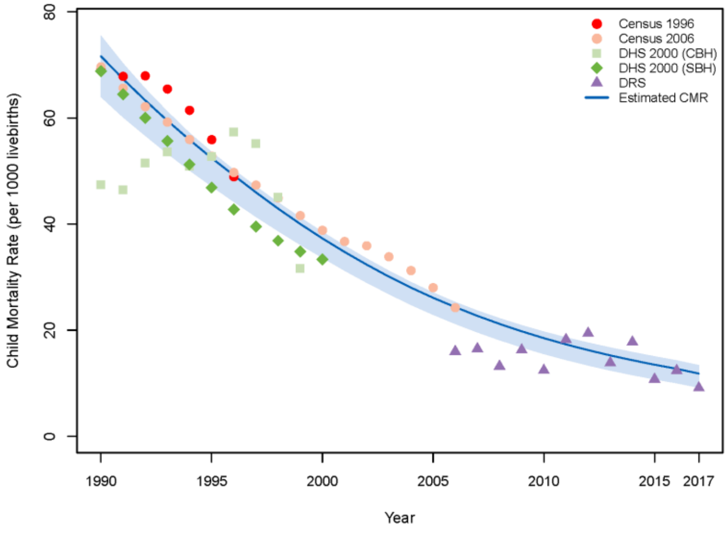

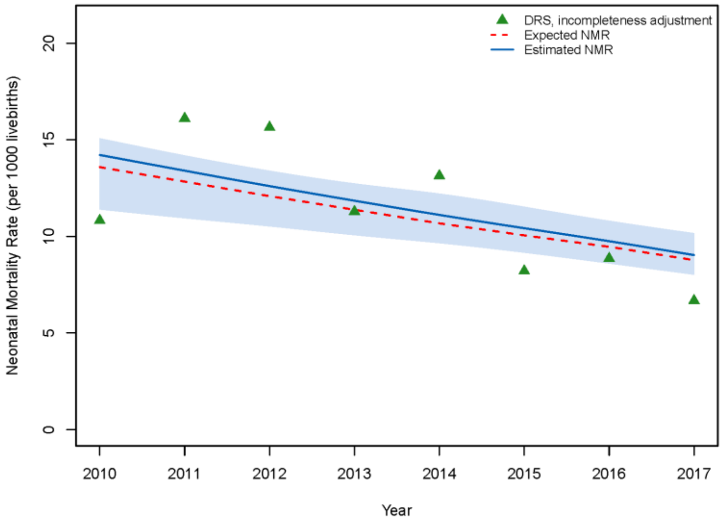

Ilam

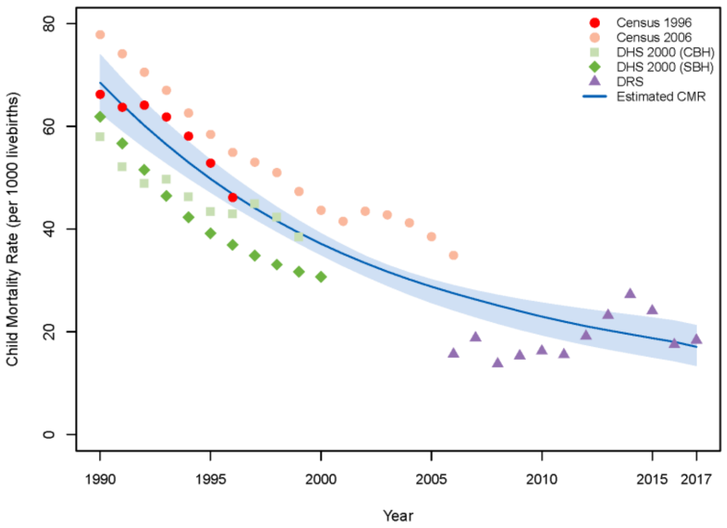

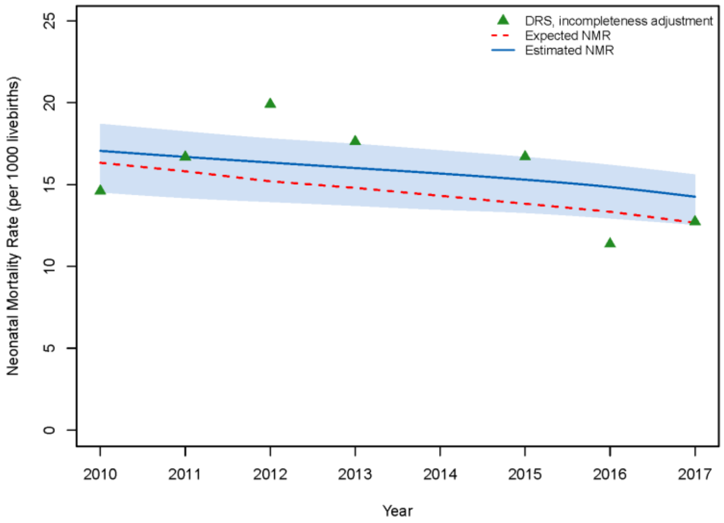

Kohgiluyeh and Boyer-Ahmad

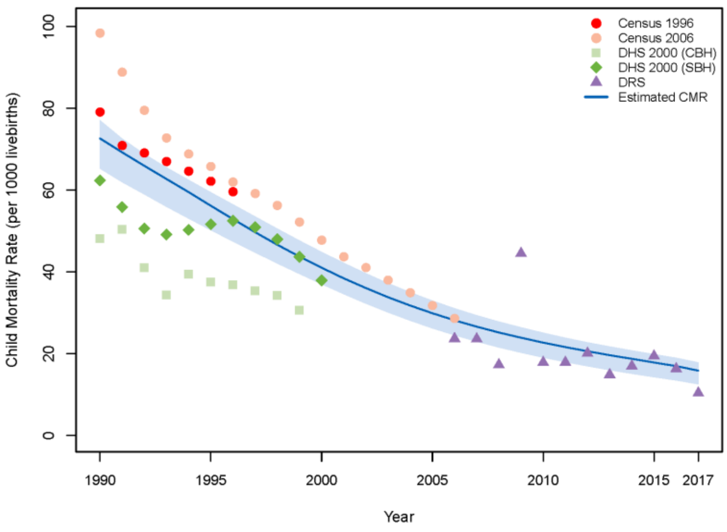

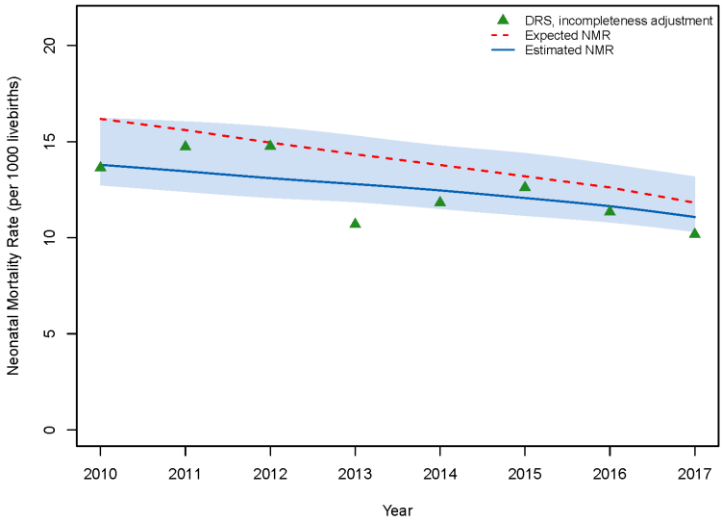

Bushehr

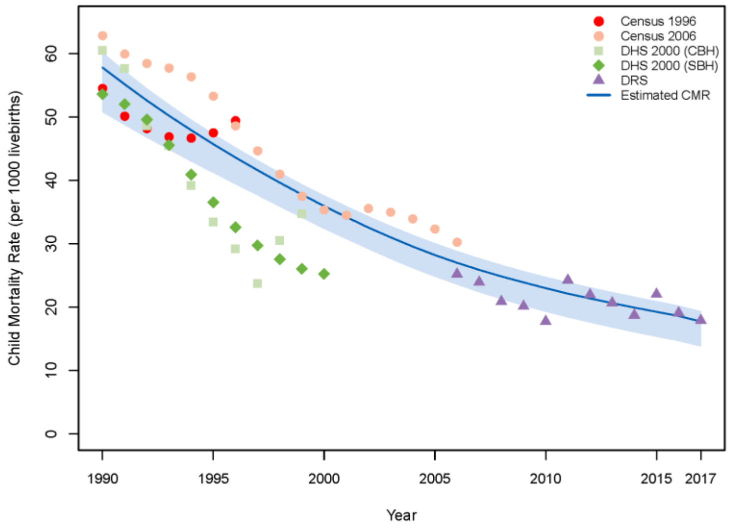

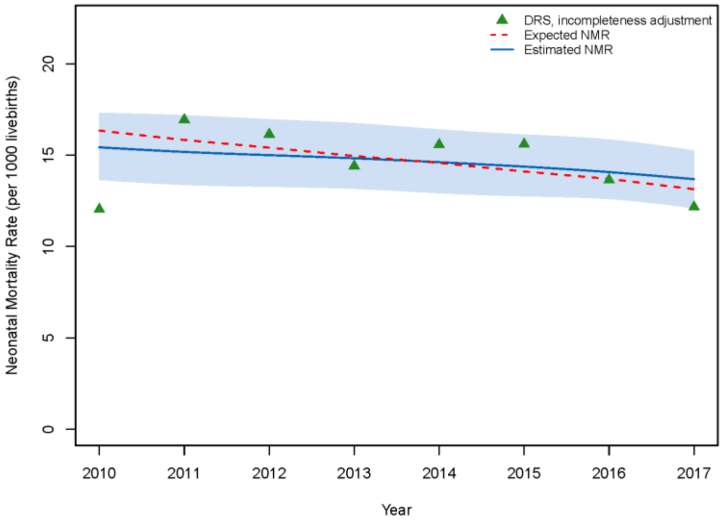

Zanjan

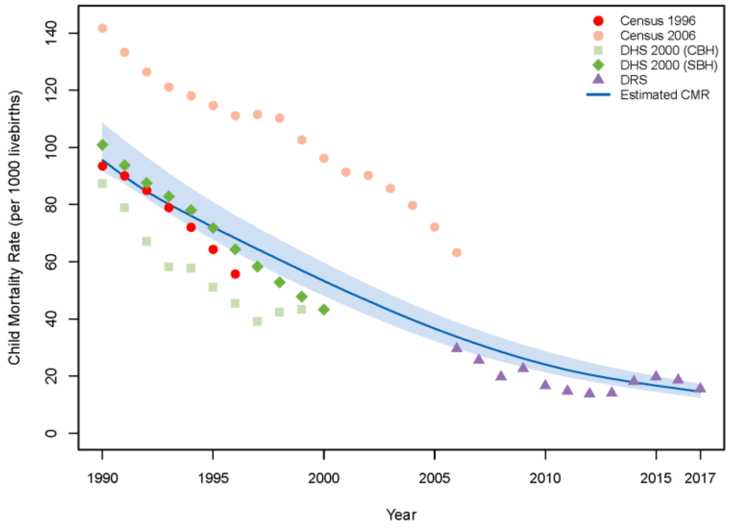

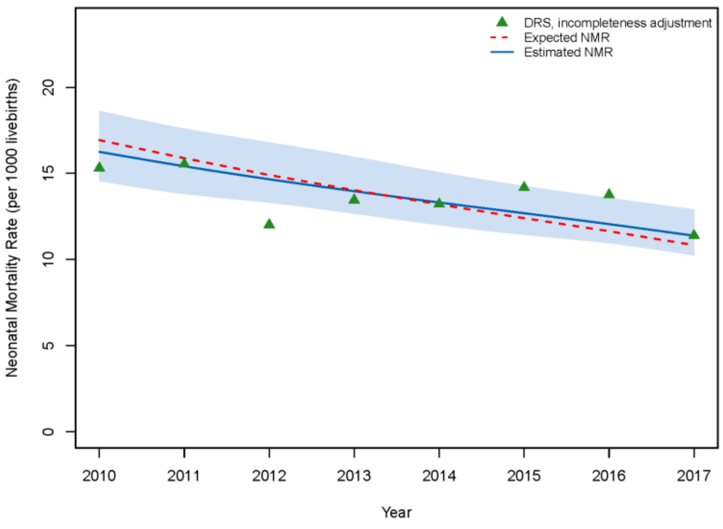

Semnan

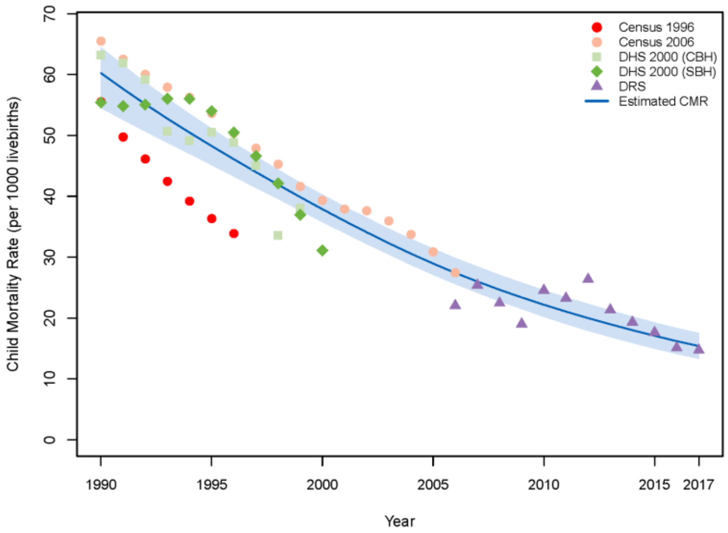
Census 1996
Census 2006
DHS 2000 (CBH)
DHS 2000 (SBH)
DRS
Estimated CMR
Child Mortality Rate (per 1000 livebirths)
Year
0
10
20
30
40
50
60
70
1990
1995
2000
2005
2010
2015
2017

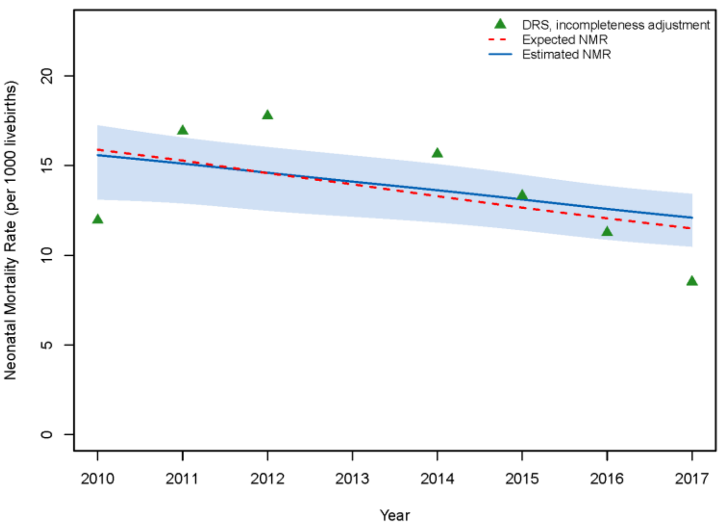
DRS, incompleteness adjustment
Expected NMR
Estimated NMR
Neonatal Mortality Rate (per 1000 livebirths)
Year
0
5
10
15
20
2010
2011
2012
2013
2014
2015
2016
2017

Yazd

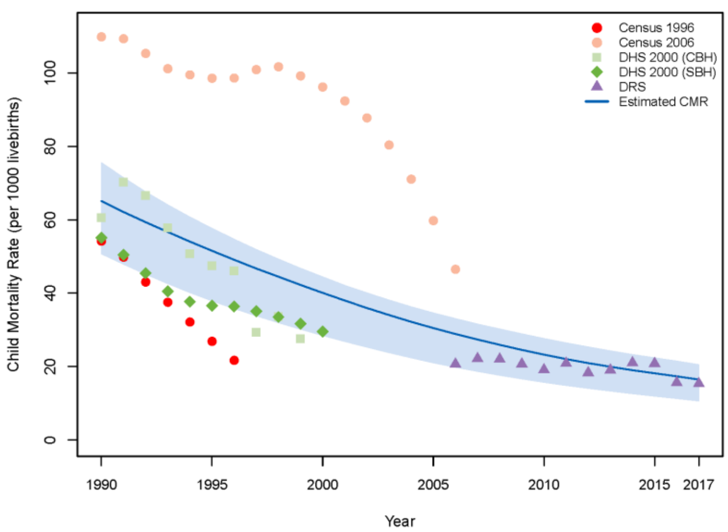
Census 1996
Census 2006
DHS 2000 (CBH)
DHS 2000 (SBH)
DRS
Estimated CMR
Child Mortality Rate (per 1000 livebirths)
Year
0
20
40
60
80
100
1990
1995
2000
2005
2010
2015
2017

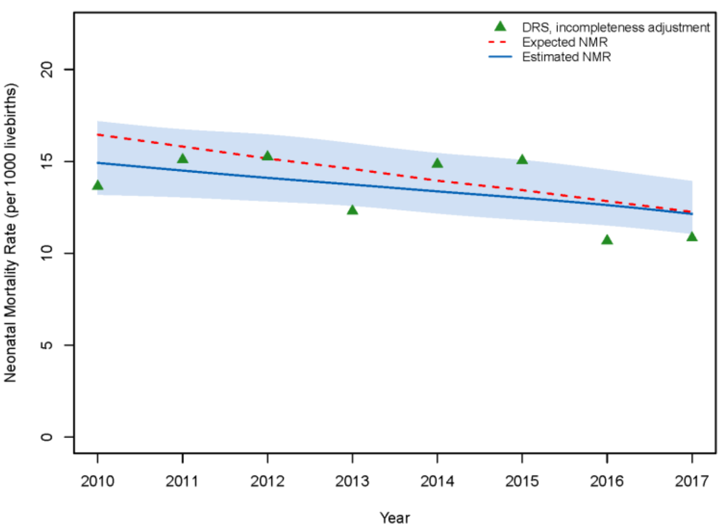
DRS, incompleteness adjustment
Expected NMR
Estimated NMR
Neonatal Mortality Rate (per 1000 livebirths)
Year
0
5
10
15
20
2010
2011
2012
2013
2014
2015
2016
2017

Hormozgan

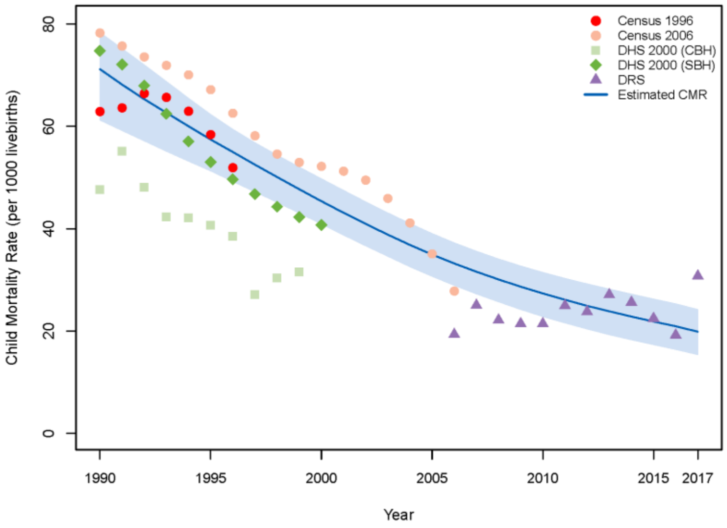

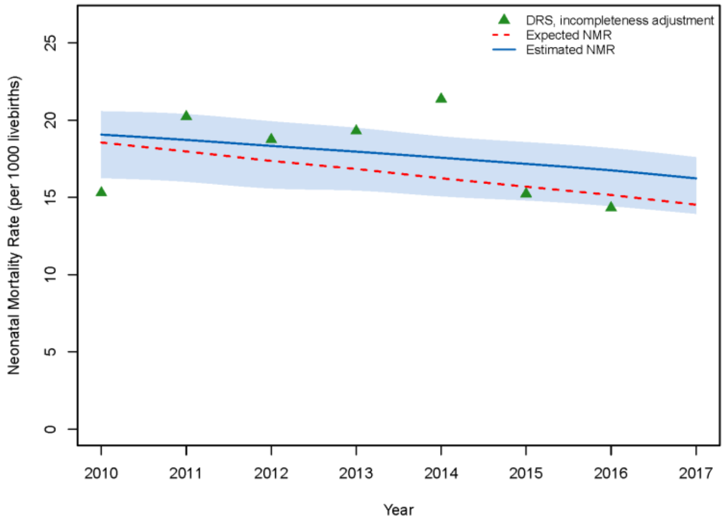

Tehran

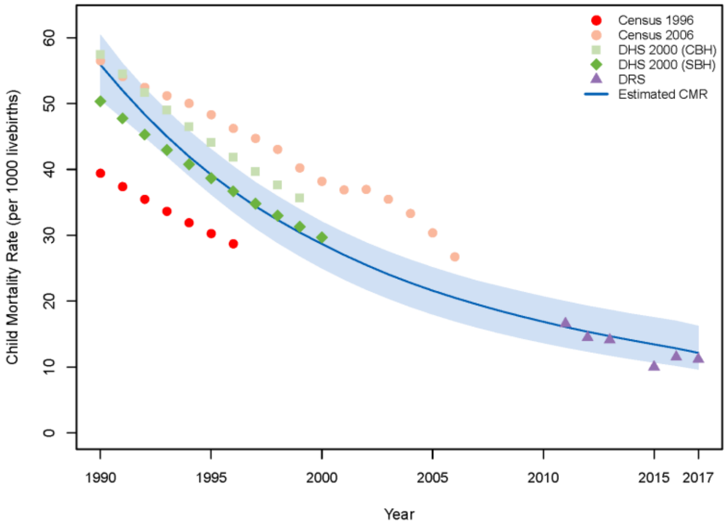

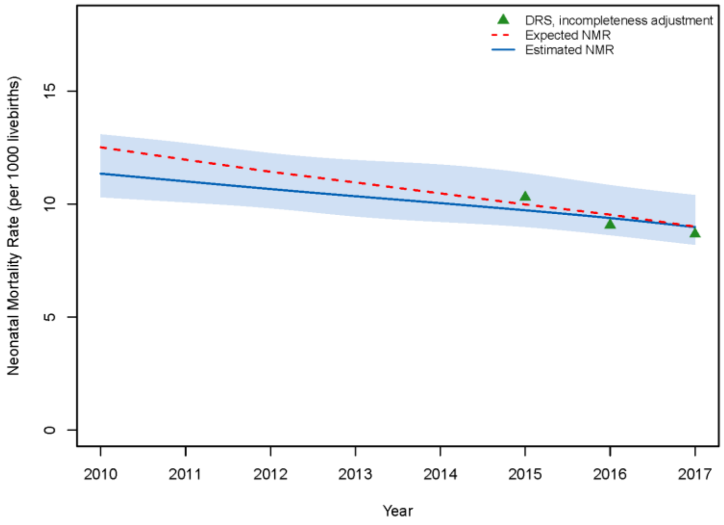

Ardabil

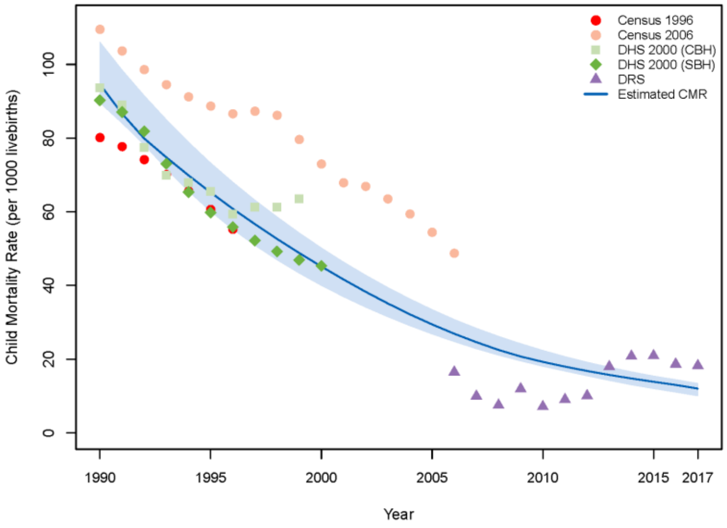
Census 1996
Census 2006
DHS 2000 (CBH)
DHS 2000 (SBH)
DRS
Estimated CMR
Child Mortality Rate (per 1000 livebirths)
Year

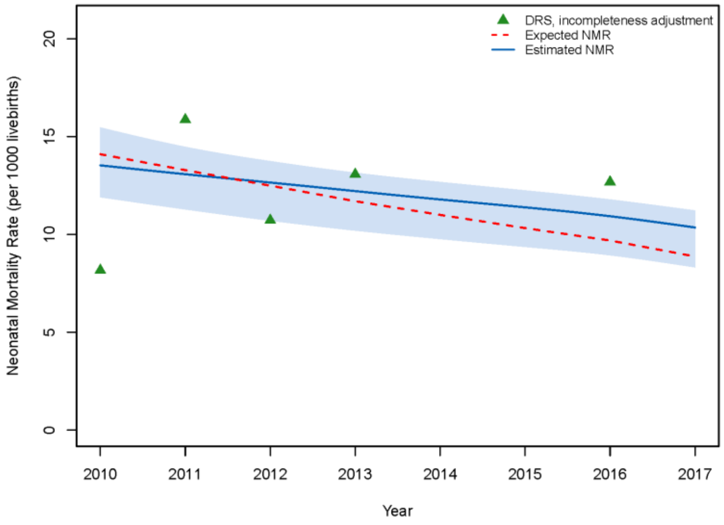
DRS, incompleteness adjustment
Expected NMR
Estimated NMR
Neonatal Mortality Rate (per 1000 livebirths)
Year

Qom

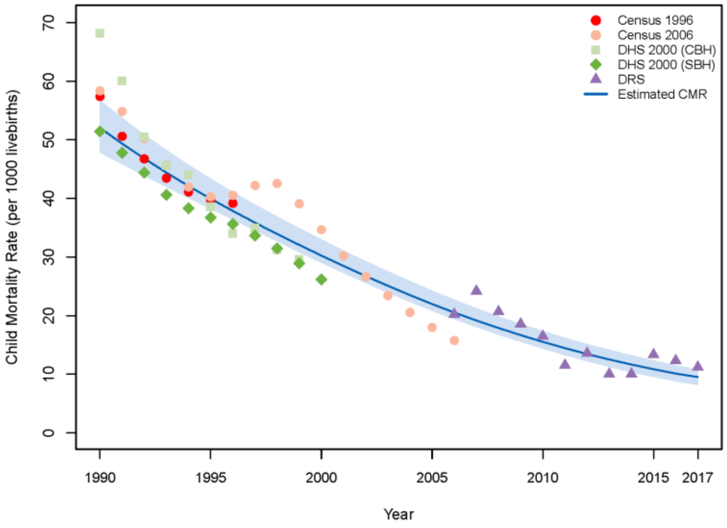
Census 1996
Census 2006
DHS 2000 (CBH)
DHS 2000 (SBH)
DRS
Estimated CMR
Child Mortality Rate (per 1000 livebirths)
Year

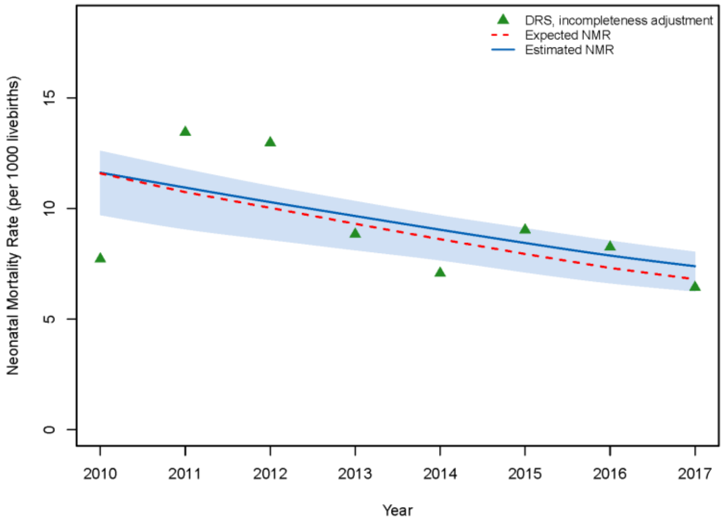
DRS, incompleteness adjustment
Expected NMR
Estimated NMR
Neonatal Mortality Rate (per 1000 livebirths)
Year

Qazvin

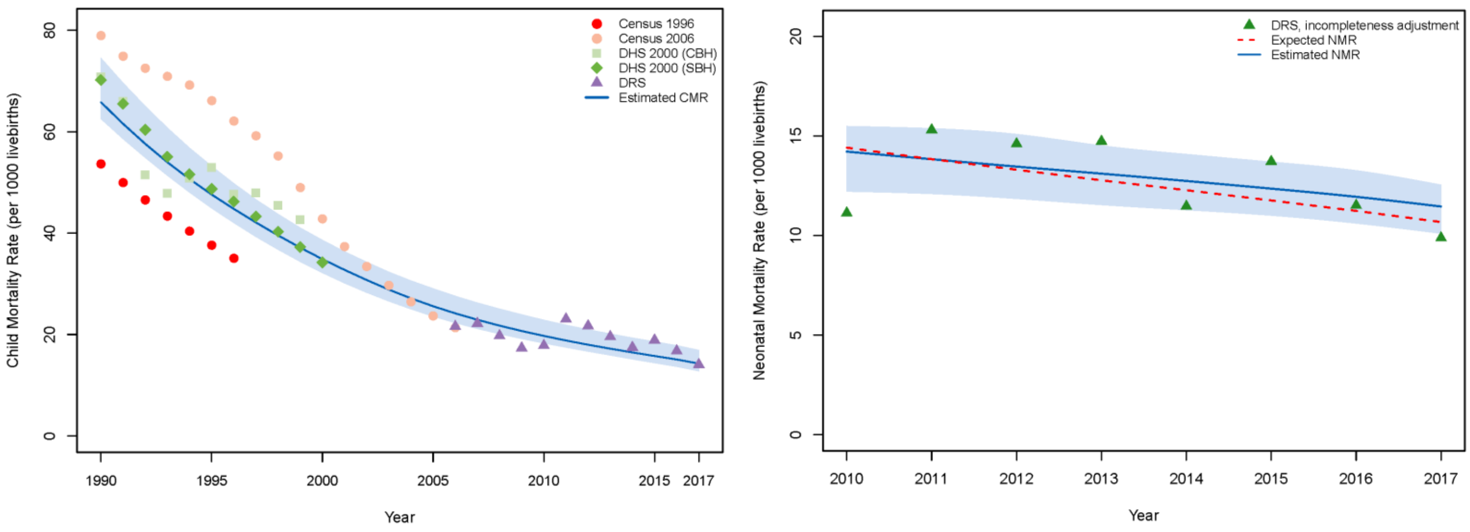

Golestan

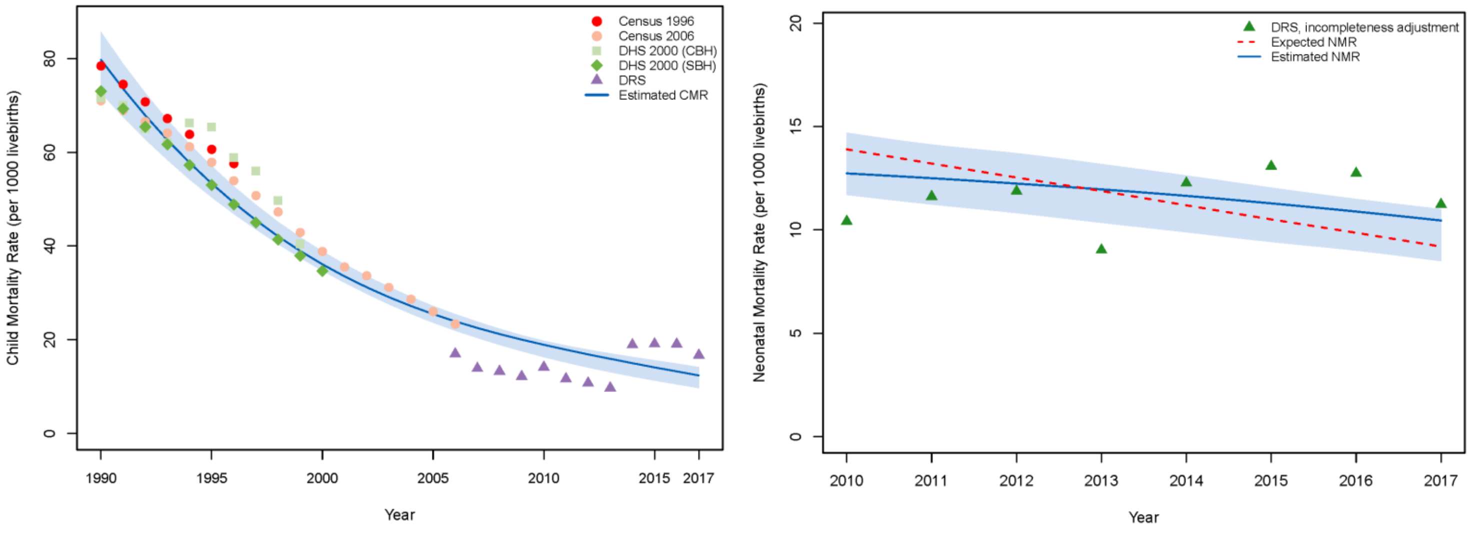

Khorasan, North

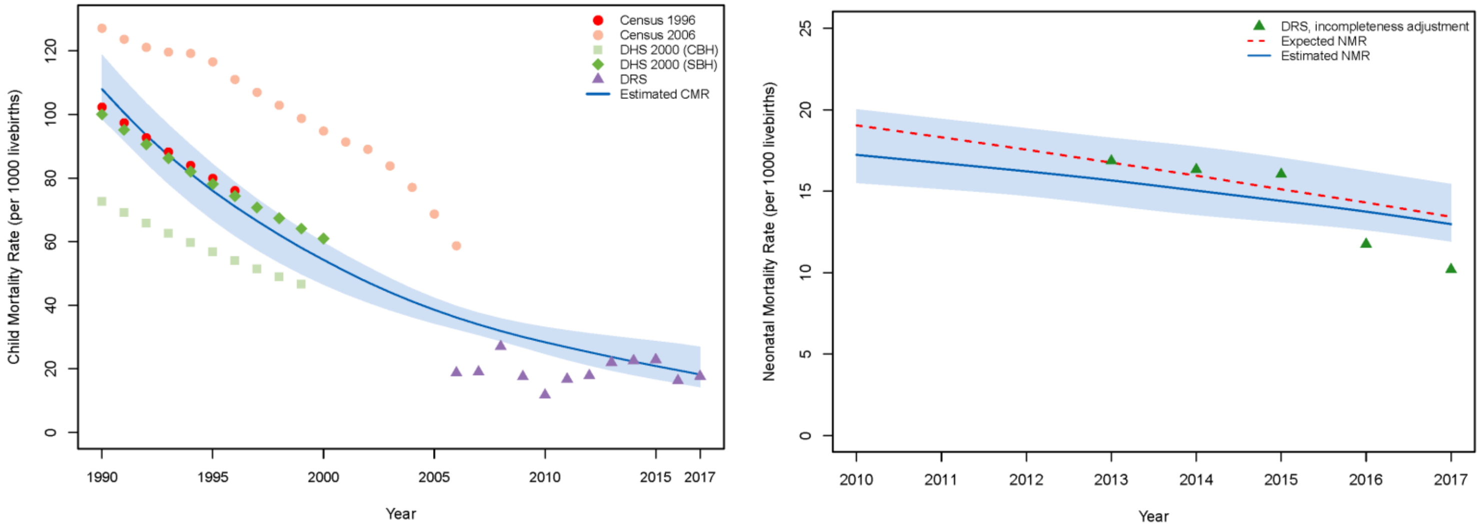

Khorasan, South

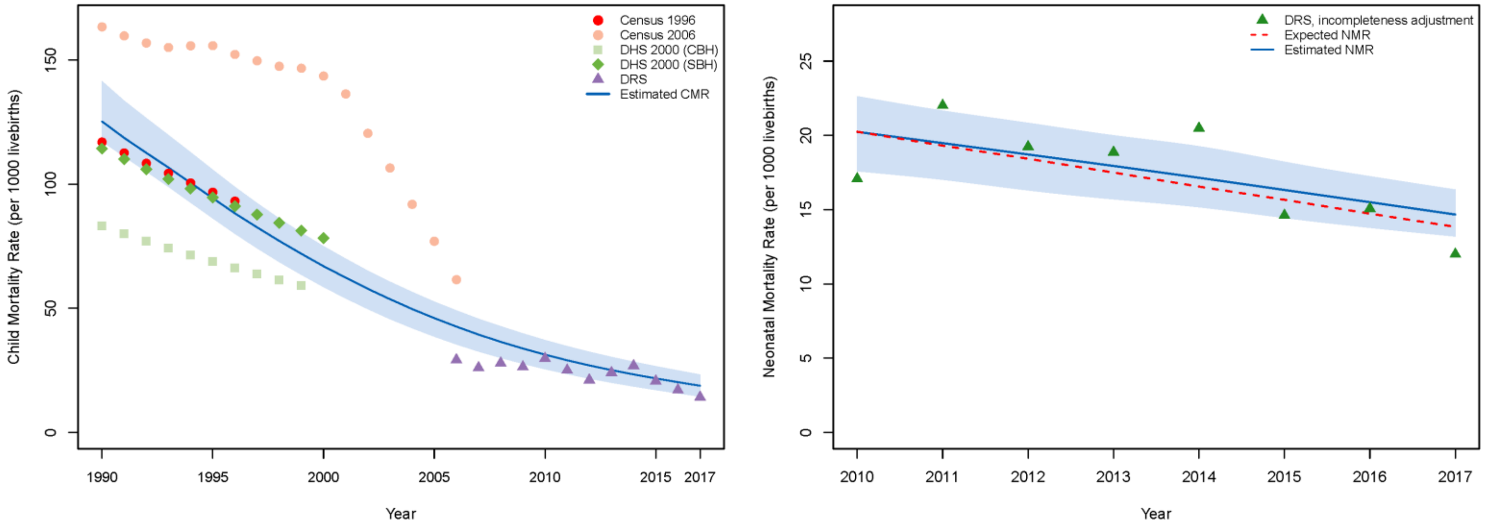

Alborz

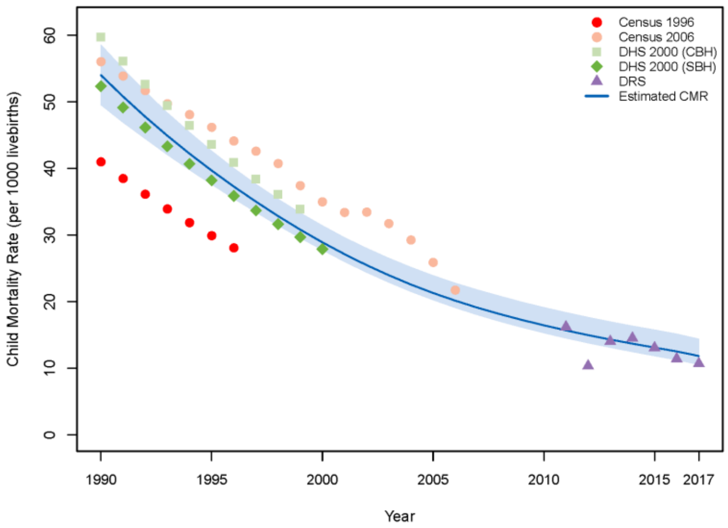

Census 1996
Census 2006
DHS 2000 (CBH)
DHS 2000 (SBH)
DRS
Estimated CMR
Child Mortality Rate (per 1000 livebirths)
Year

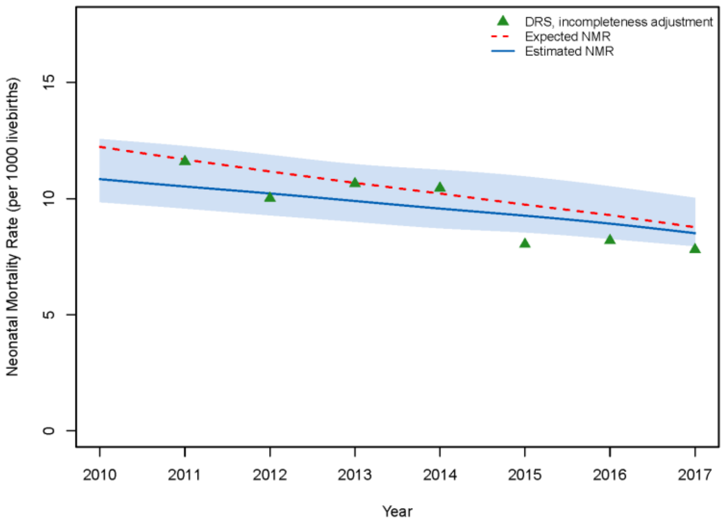

DRS, incompleteness adjustment
Expected NMR
Estimated NMR
Neonatal Mortality Rate (per 1000 livebirths)
Year

5.2. Projection trends of NMR from 2017 to 2030 for Iranian provinces

Markazi

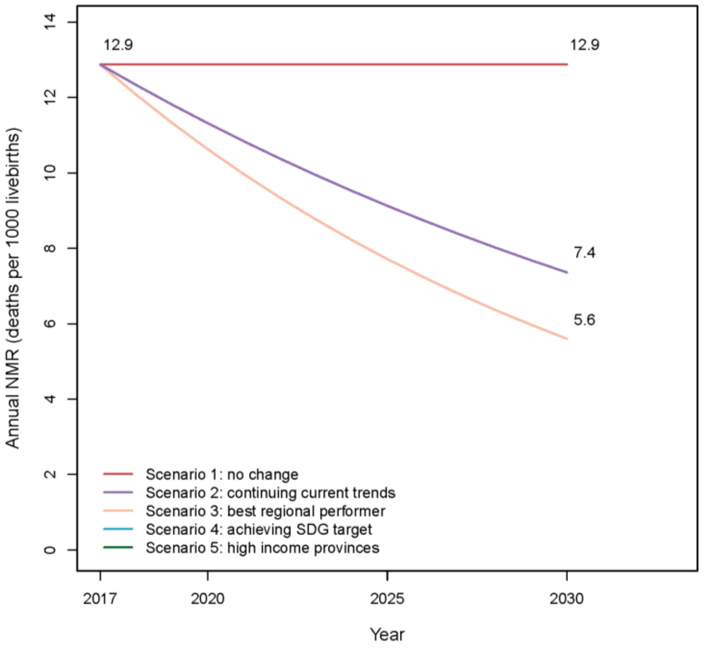

Gilan

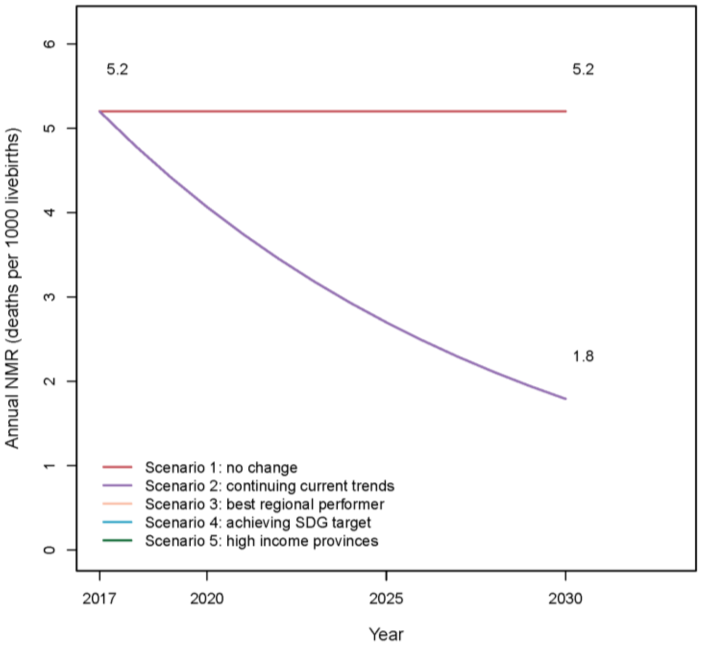

Mazandaran

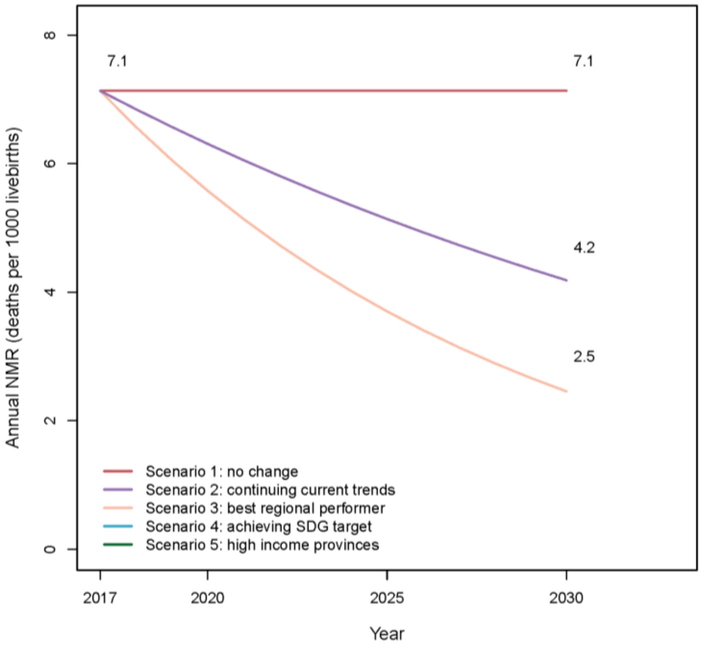

Azerbaijan, East

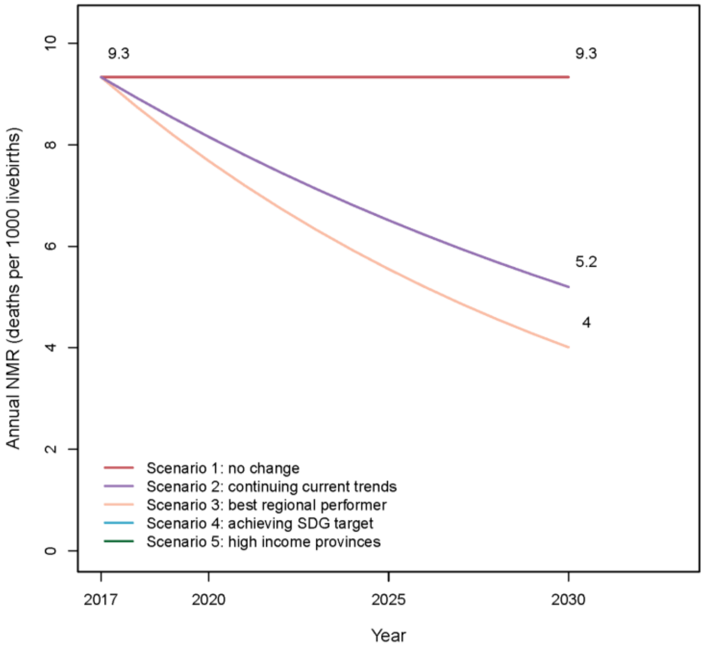

Azerbaijan, West

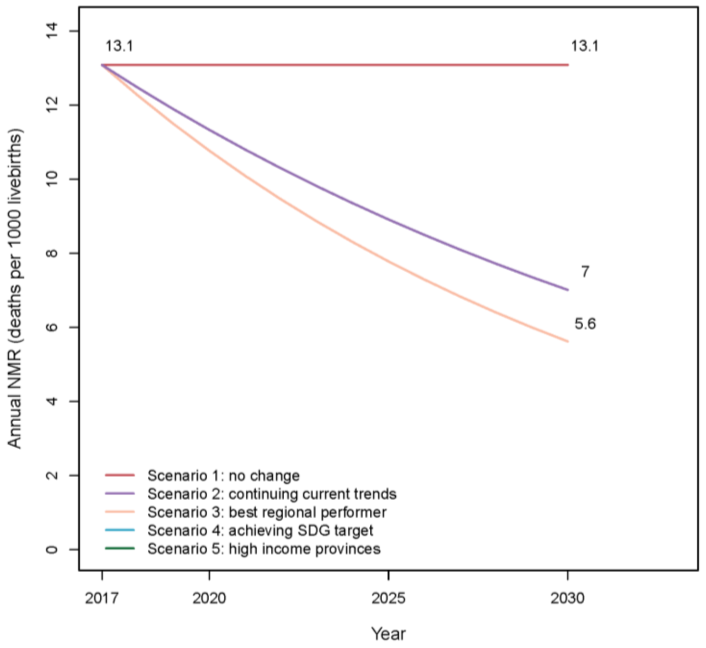

Kermanshah

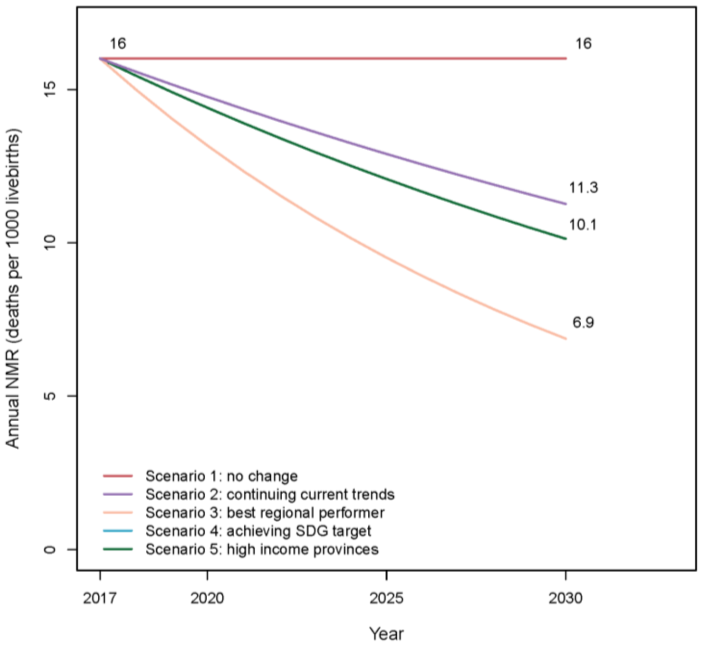

Khuzestan

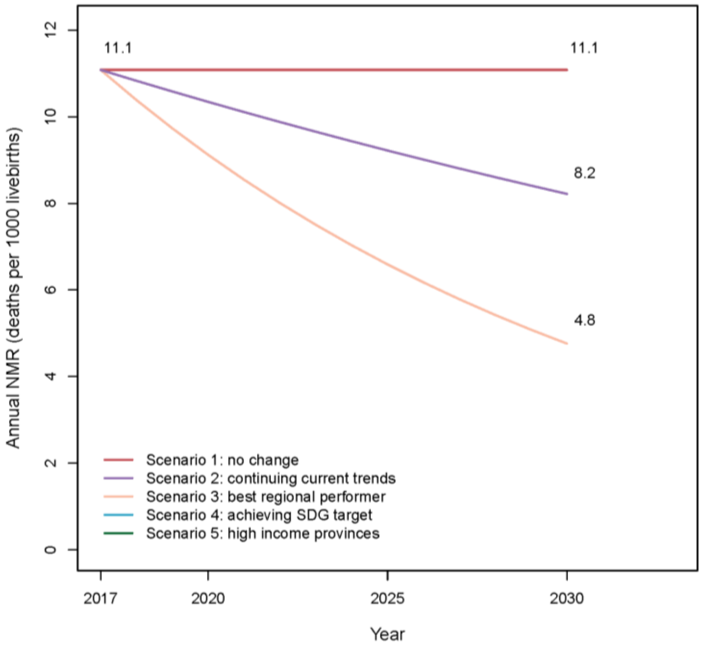

Fars

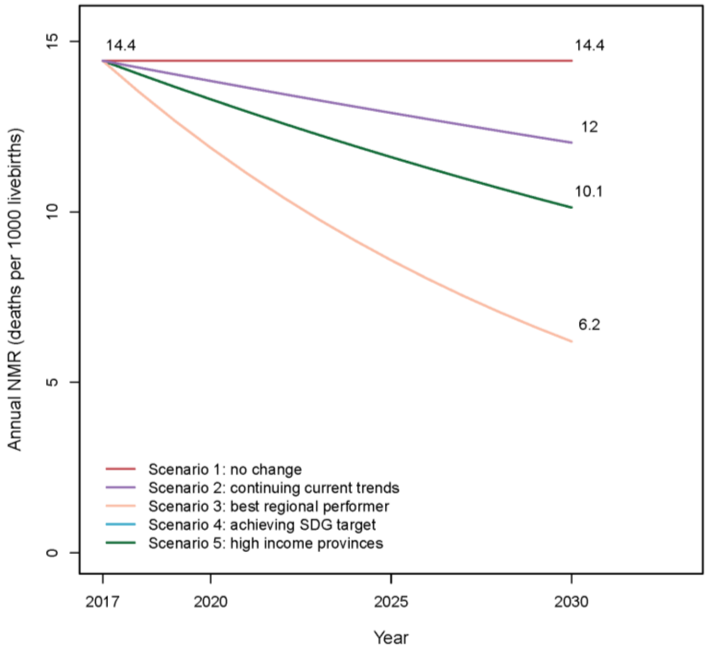

Kerman

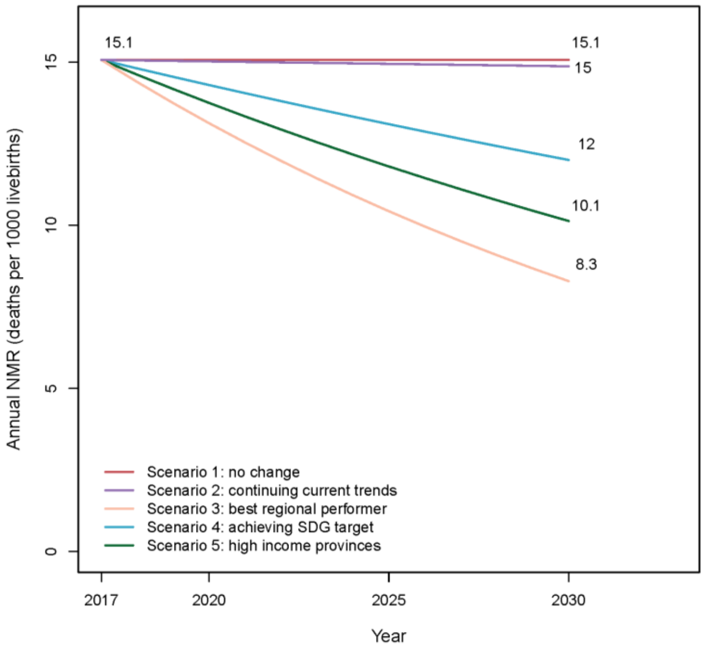

Khorasan, Razavi

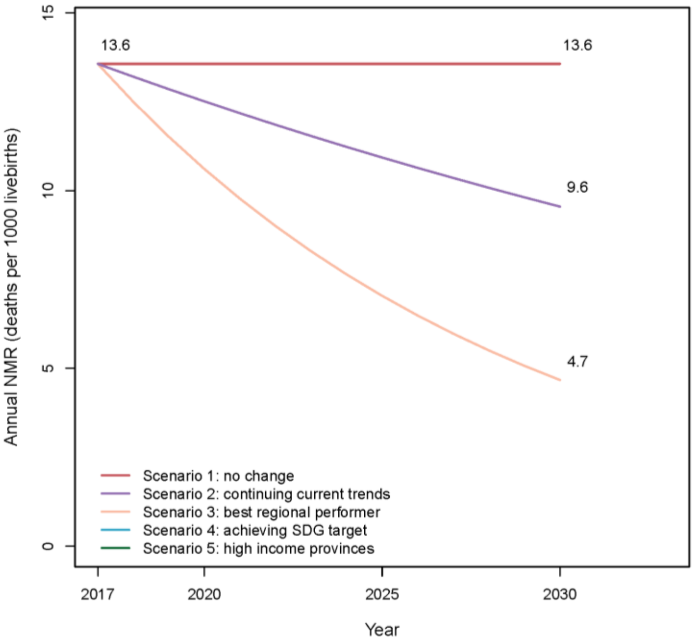

Isfahan

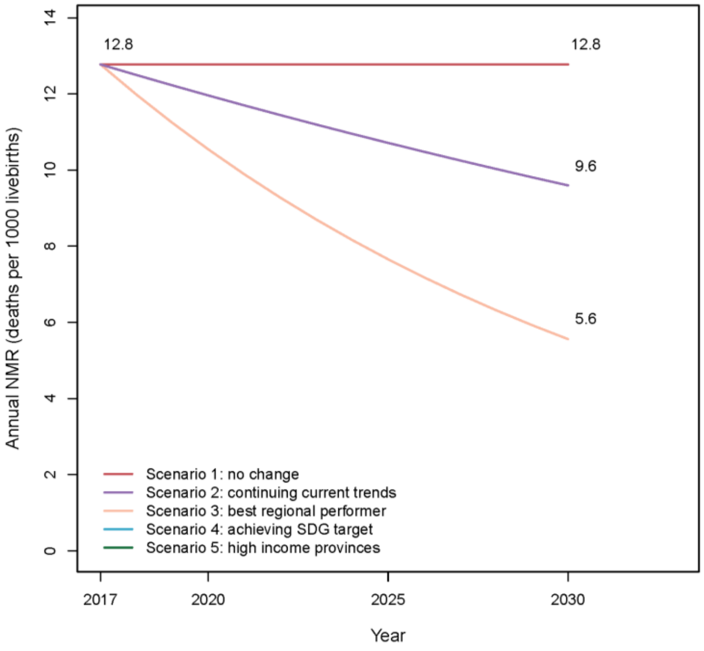

Sistan and Baluchistan

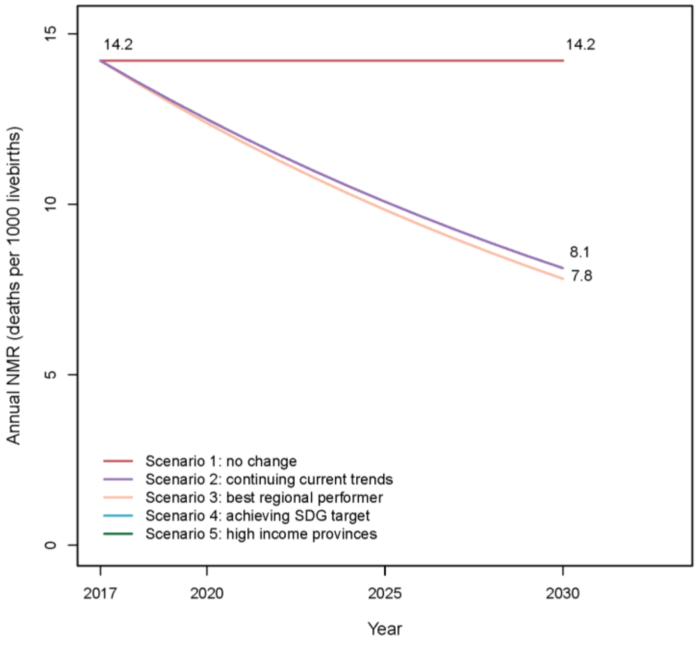

Kordestan

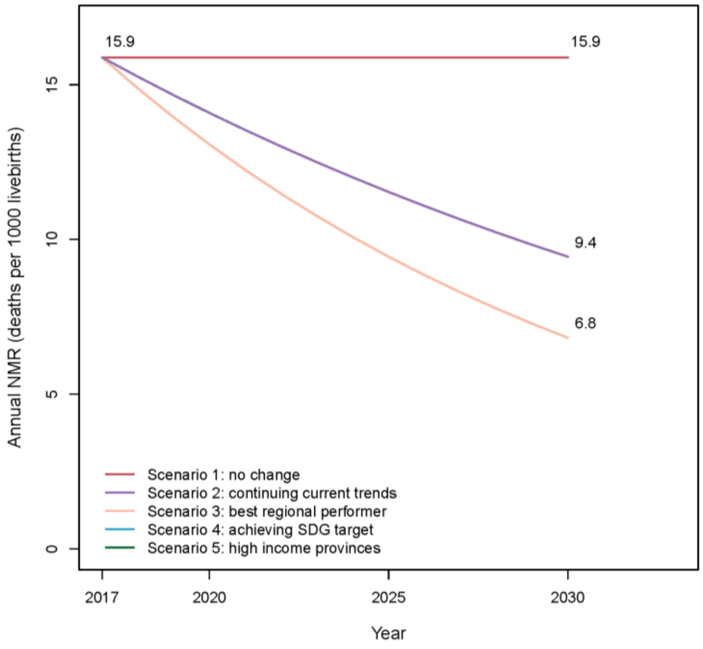

Hamadan

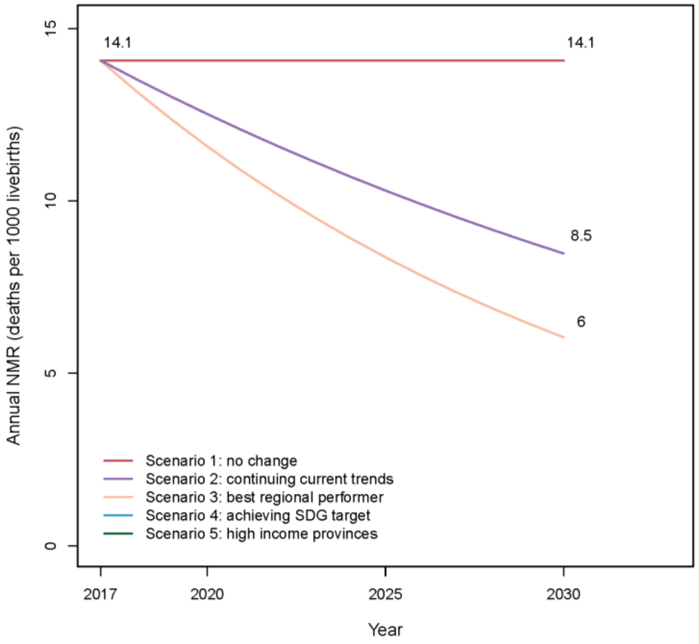

Chahar Mahaal and Bakhtiari

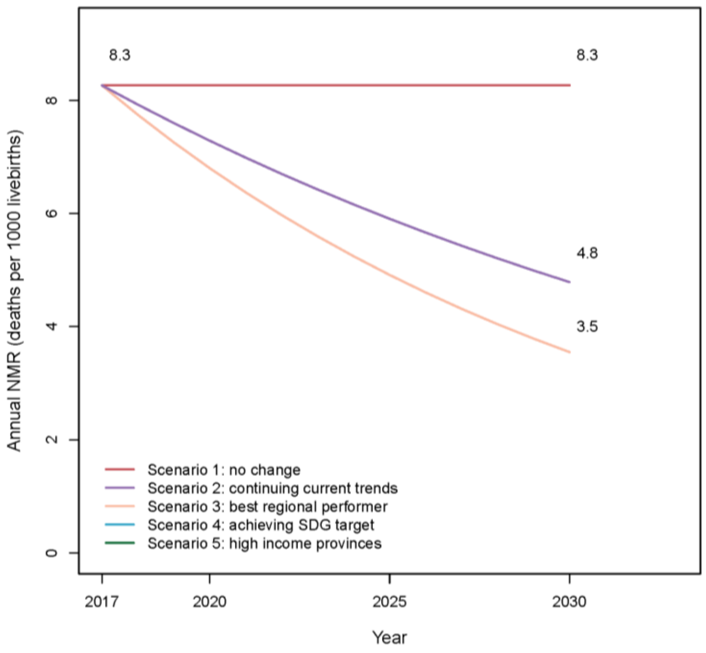

Lorestan

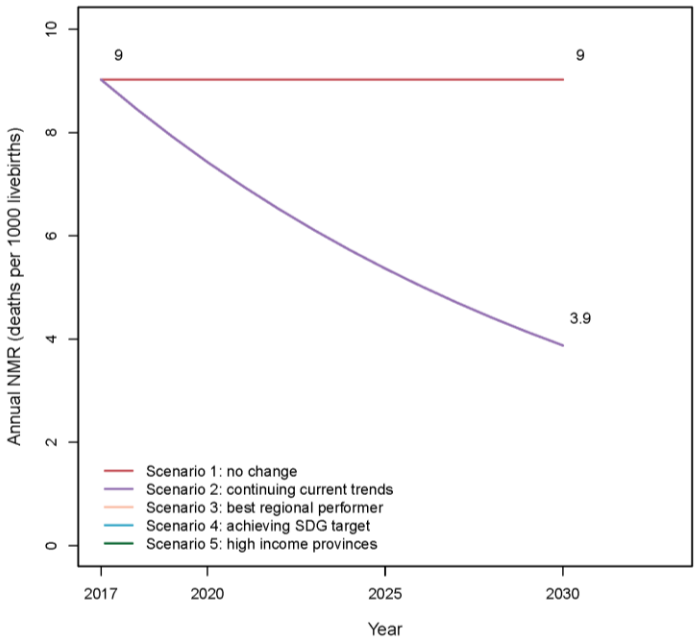

Ilam

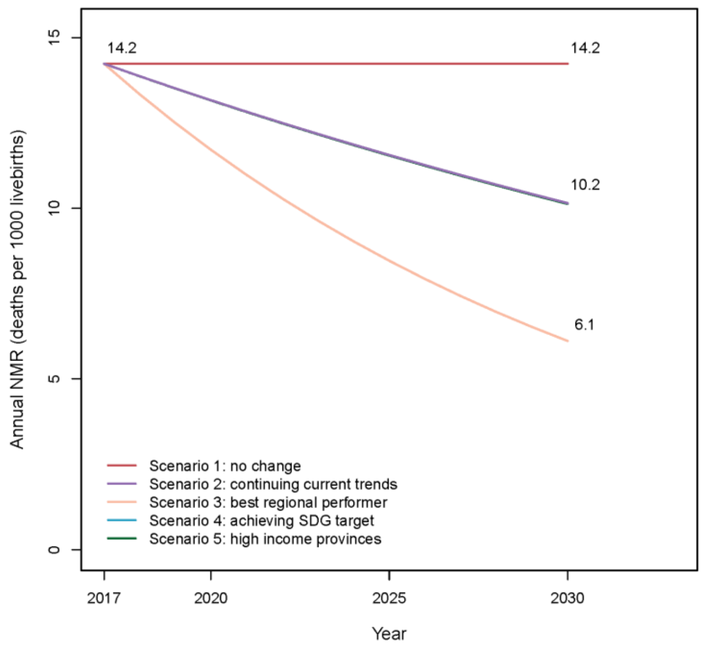

Kohgiluyeh and Boyer-Ahmad

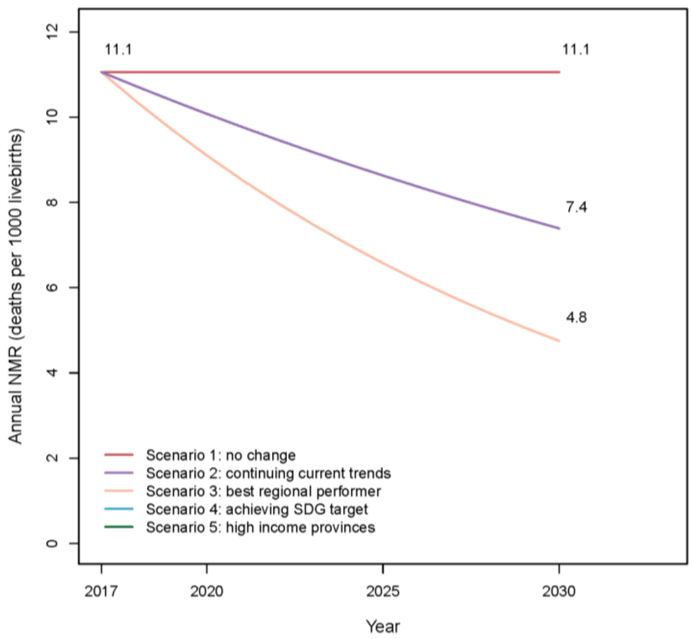

Bushehr

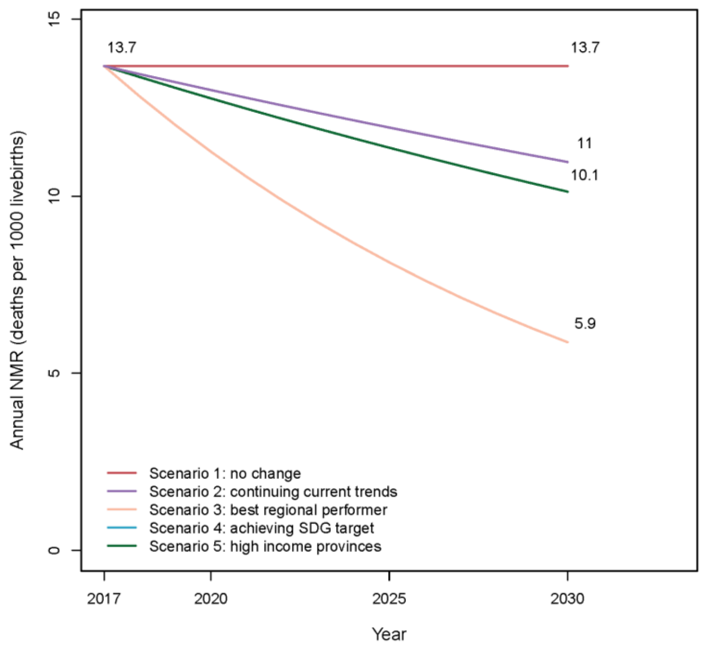

Zanjan

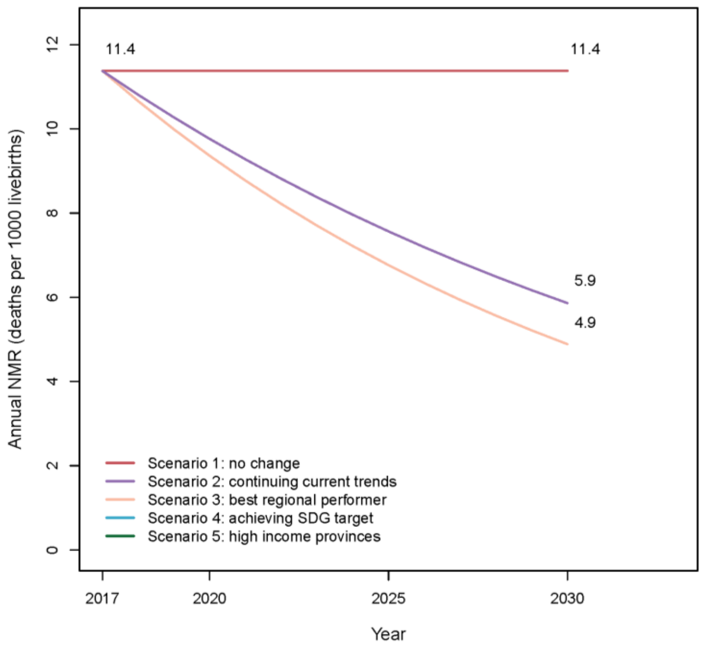

Semnan

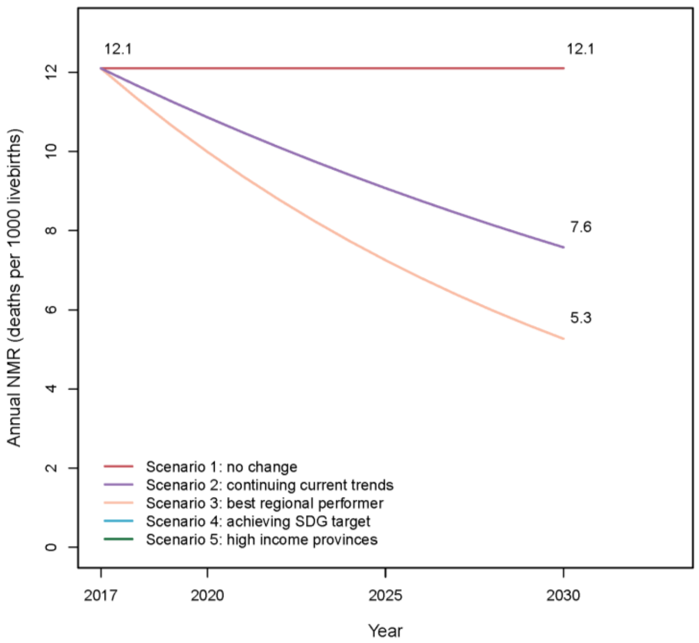

Yazd

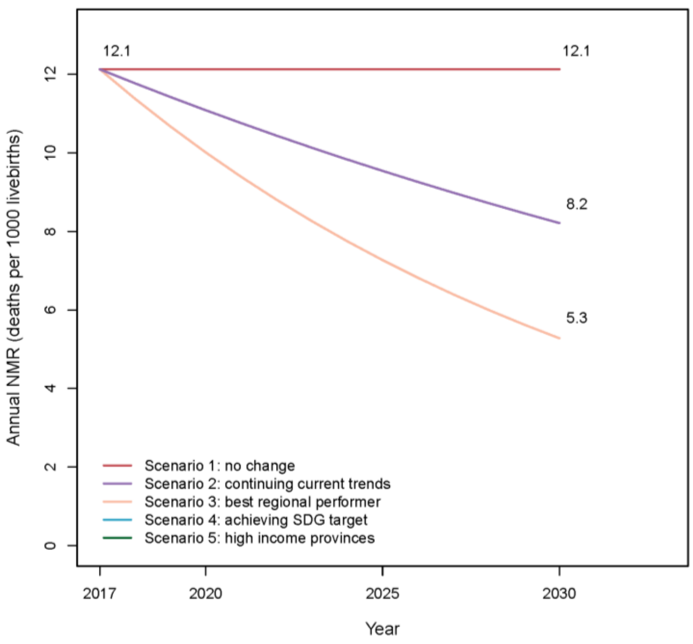

Hormozgan

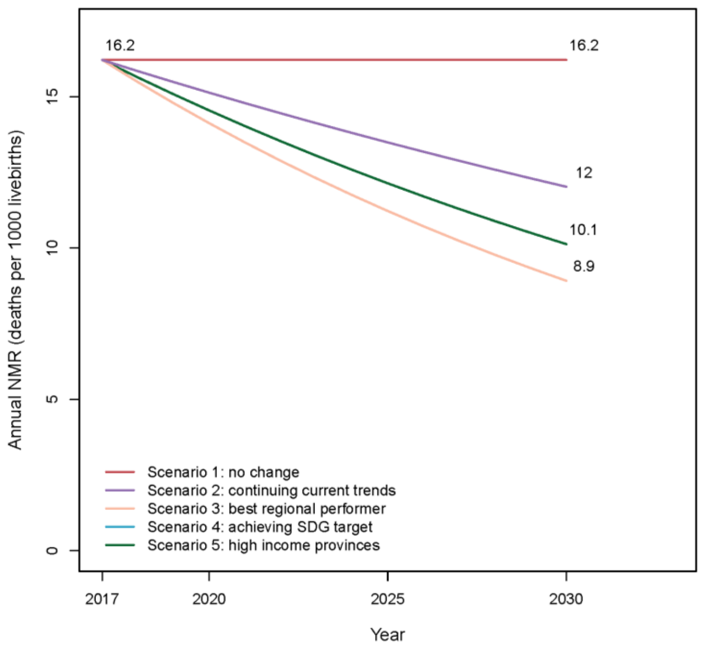

Tehran

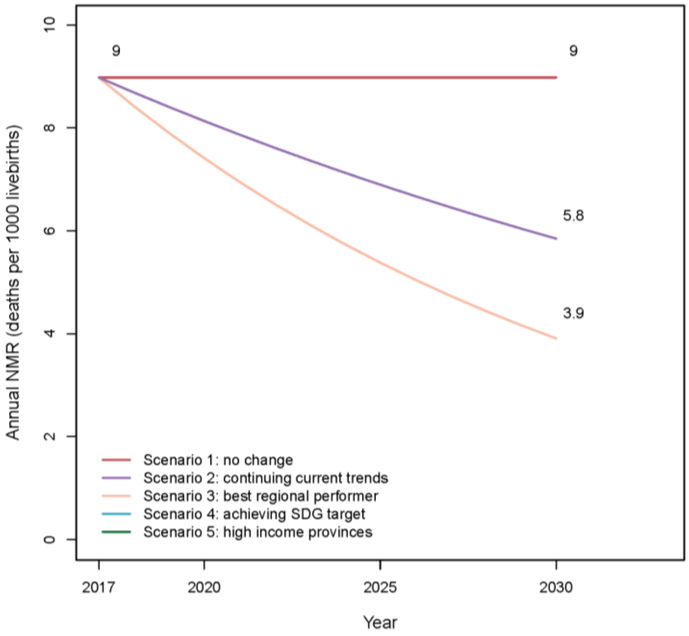

Ardabil

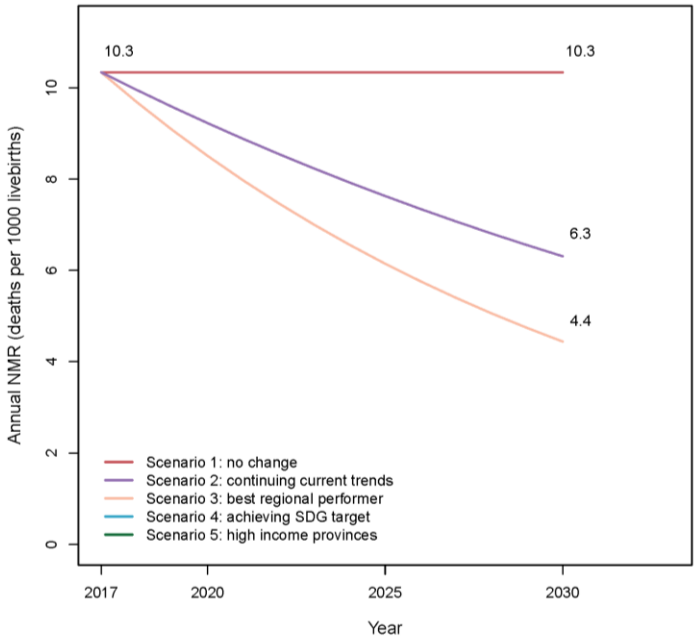

Qom

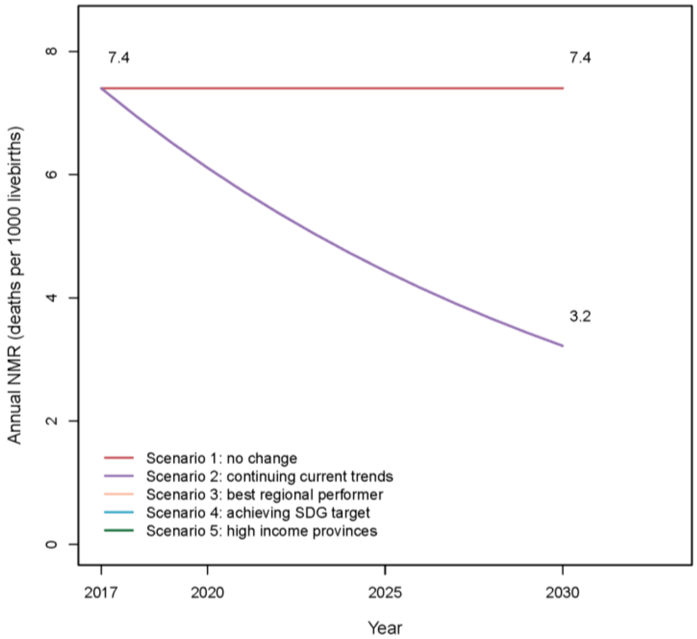

Qazvin

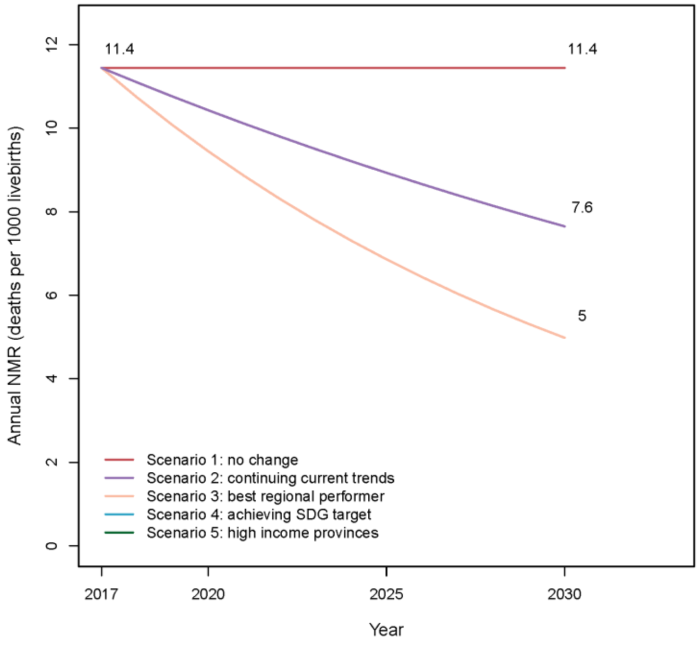

Golestan

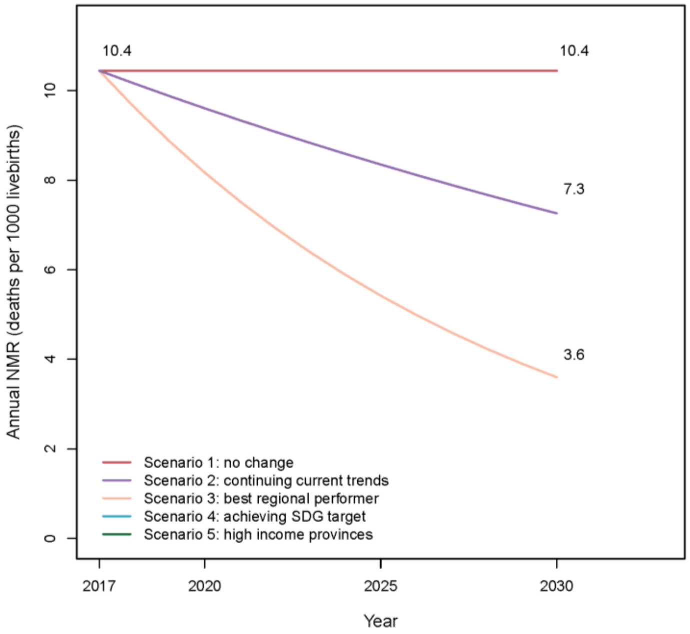

Khorasan, North

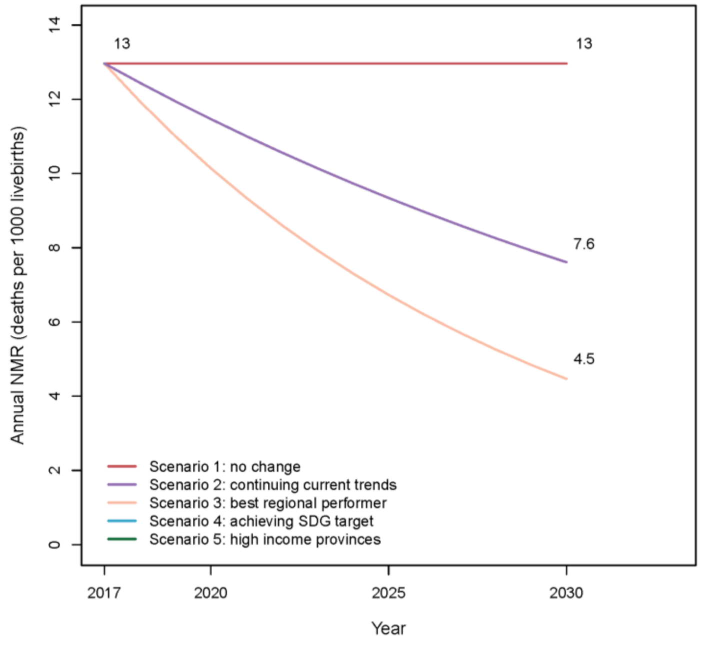

Khorasan, South

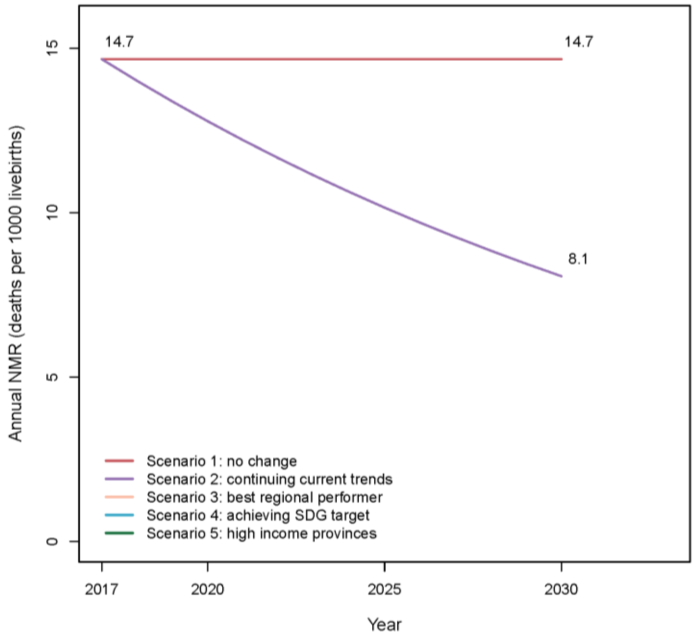

Alborz

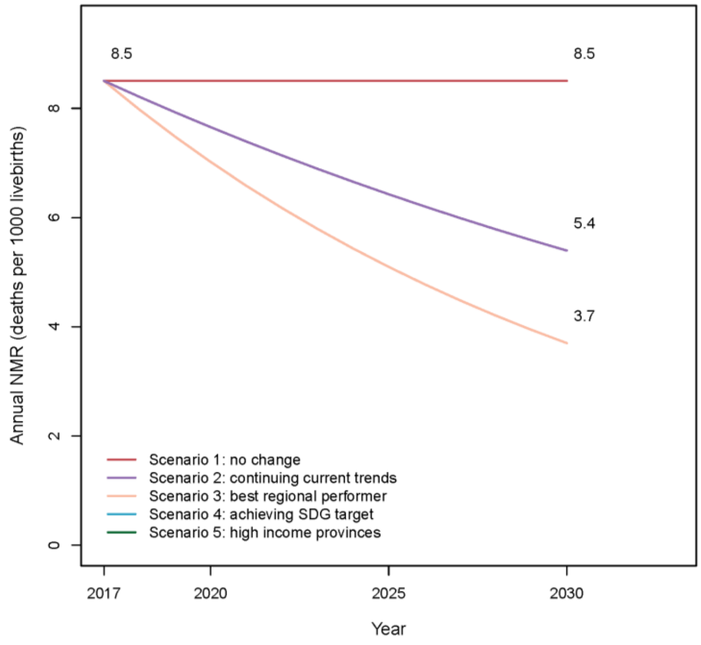

6. References

1. Mohammadi Y, Parsaeian M, Mehdipour P, Khosravi A, Larijani B, Sheidaei A, et al. Measuring Iran's success in achieving Millennium Development Goal 4: a systematic analysis of under-5 mortality at national and subnational levels from 1990 to 2015. The Lancet Global Health. 2017;5(5):e537-e44.
2. Rashidian A, Karimi-Shahanjarini A, Khosravi A, Elahi E, Beheshtian M, Shakibazadeh E, et al. Iran's multiple indicator demographic and health survey-2010: Study protocol. International journal of preventive medicine. 2014;5(5):632.
3. Jafari N, Kabir M, Motlagh M. Death registration system in IR Iran. 2009.
4. Rajaratnam JK, Tran LN, Lopez AD, Murray CJ. Measuring under-five mortality: validation of new low-cost methods. PLoS Med. 2010;7(4):e1000253.
5. Economic UNDoI, Population NRCCo. Indirect techniques for demographic estimation: New York: United Nations; 1983.
6. Guillot M. Tools for demographic estimation. Taylor & Francis; 2015.
7. Honaker J, King G, Blackwell M. Amelia II: A program for missing data. Journal of statistical software. 2011;45(7):1-47.
8. Parsaeian M, Farzadfar F, Zeraati H, Mahmoudi M, Rahimighazikalayeh G, Navidi I, et al. Application of spatio-temporal model to estimate burden of diseases, injuries and risk factors in Iran 1990–2013 (study protocol). 2014.
9. Rasmussen CE, editor Gaussian processes in machine learning. Summer School on Machine Learning; 2003: Springer.
10. Mehdipour P, Navidi I, Parsaeian M, Mohammadi Y, MORADI LM, REZAEI DE, et al. Application of Gaussian Process Regression (GPR) in estimating under-five mortality levels and trends in Iran 1990-2013, study protocol. 2014.
11. Foreman KJ, Lozano R, Lopez AD, Murray CJ. Modeling causes of death: an integrated approach using CODEm. Population health metrics. 2012;10(1):1.
12. Blangiardo M, Cameletti M. Spatial and spatio-temporal Bayesian models with R-INLA: John Wiley & Sons; 2015.
13. Kontis V, Bennett JE, Mathers CD, Li G, Foreman K, Ezzati M. Future life expectancy in 35 industrialised countries: projections with a Bayesian model ensemble. The Lancet. 2017;389(10076):1323-35.
14. Alexander M, Alkema L. Global estimation of neonatal mortality using a Bayesian hierarchical splines regression model. Demographic Research. 2018;38:335-72.
